# Mechanistic insights into periodontal ligament stem cell-derived exosomes in tissue regeneration

**DOI:** 10.1007/s00784-025-06422-1

**Published:** 2025-06-25

**Authors:** Paras Ahmad, Nathan Estrin, Nima Farshidfar, Yufeng Zhang, Richard J. Miron

**Affiliations:** 1 Department of Research, Advanced PRF Education, Jupiter, Florida USA; 2 Lake Erie College of Osteopathic Medicine School of Dental Medicine, Bradenton, Florida USA; 3 Department of Periodontology, University of Bern, Bern, Switzerland; 4 Department of Oral Implantology, University of Wuhan, Wuhan, China

**Keywords:** Exosomes, Periodontal ligament, Periodontium, Regeneration, Tissue engineering

## Abstract

**Objective:**

This review comprehensively examined the emerging role of exosomes derived from periodontal ligament stem cells (PDLSC-Exos) in regenerative medicine and dentistry, with a particular focus on their therapeutic potential in periodontitis—a prevalent inflammatory disease characterized by the progressive destruction of periodontal tissues.

**Methods:**

An initial search on Scopus, Web of Science, and PubMed using terms associated with exosomes (i.e., exosomes, exosomal, extracellular vesicles, and EVs) and periodontal ligament stem cells without any limitation of publication year and field of study was performed on October 31st, 2024.

**Results:**

PDLSC-Exos demonstrated significant therapeutic efficacy in both bone and periodontal regeneration as well as various medical conditions through the enhancement of cellular proliferation, osteoblast differentiation, and the modulation of inflammatory responses. These exosomes function by regulating miRNA and activating essential signaling pathways, thereby facilitating periodontal/bone regeneration, angiogenesis, and tissue repair in disorders such as periodontitis, OTM, and skeletal bony defects. Furthermore, they exhibited anti-inflammatory properties, leading to improved outcomes under inflammatory conditions such as periodontitis, IBD, and MS. Moreover, PDLSC-Exos played a role in anti-microbial and anti-cancer interventions, demonstrating their diverse applicability.

**Conclusion:**

The cell-free nature of these therapeutic agents makes PDLSC-Exos a versatile and promising tool for regenerative medicine and immune system regulation. The cell-free nature of these therapeutic agents positions PDLSC-Exos as a promising and adaptable instrument for regenerative medicine and immune system modulation.

**Clinical relevance:**

PDLSC-Exos offer a promising acellular therapy for periodontal regeneration, overcoming limitations of cell-based approaches by enhancing tissue repair, modulating inflammation, and improving clinical translation in regenerative medicine and dentistry.

**Supplementary Information:**

The online version contains supplementary material available at 10.1007/s00784-025-06422-1.

## Introduction

Bone constitutes a specialized form of connective tissue that establishes the structural framework of the skeletal system. It comprises mineralized deposits, collagen fibers, and cells [1]. It plays a crucial role in providing support, protection, and facilitating movement within the body, while simultaneously serving as a locus for hematopoiesis and mineral reservoir. Bone fulfills numerous functions, including offering mechanical support to the organism, establishing attachment sites for muscles, ligaments, and tendons, and functioning as a protective barrier for various tissues [2]. Of particular importance, hematopoiesis and the provision of essential minerals by the bone marrow, underscoring the critical nature of bone regeneration in response to damage incurred from diverse pathological conditions [3]. Presently, the incidence of bone injuries and diseases is escalating owing to a multitude of factors stemming from traumatic events, neoplasms, inflammatory and degenerative disorders, and infections [4]. The regeneration of rigid bone tissue necessitates the synergistic interplay of mechanical and biological characteristics akin to those of the native bone extracellular matrix [5].

Periodontitis is defined by the deterioration of periodontal attachment, bleeding gums, alveolar bone resorption, and, in advanced cases, tooth mobility [6–8]. It constitutes one of the most widespread infectious diseases globally, impacting approximately 20 to 50% of the world population [9]. The conventional therapeutic approaches for periodontitis primarily encompass scaling and root planning (SRP), full-mouth debridement, and periodontal maintenance [10]. Nevertheless, these interventions do not fully restore the compromised periodontal tissues [11]; thus, it is imperative to explore innovative strategies to treat periodontitis that effectively facilitate the regeneration of periodontal tissues. At present, therapies incorporating bone substitute materials are predominantly used in clinical settings addressing alveolar bone defects; however, there remain several limitations, including insufficient bone volume gain following treatment, non-optimal biocompatibility, and a lack of regenerative cells/growth factor strategies in the majority of approaches recommended [12, 13].

The ongoing debate regarding the optimal cellular phenotype for clinical osteogenesis remains unsolved; however, it has been substantiated that cellular-based methodologies for periodontal regeneration are indeed efficacious. Cell-based strategies primarily focus on the initial phases of osseous repair, particularly when the mobilization of skeletal progenitor cells may be compromised [14]. A significant impediment to enhancing the efficacy of cellular therapies lies in the identification of suitable cell sources that can be introduced at the site of periodontal defects and possess the potential to differentiate into osteoblasts while establishing neo-vasculature [15, 16].

Mesenchymal stem cell (MSC)-based treatments have demonstrated some promise in regenerative medicine [17–19]. MSCs are characterized as multipotent stem cells with the capability of self-renewal and differentiation across various lineages [20]. Owing to their distinctive immune modulation, hematopoietic support, and differentiation potential, MSCs have gained prominence as a therapeutic approach for a plethora of diseases [21]. Like other cell-based therapies, MSCs face significant challenges that limit their broader clinical use, including logistical and operational issues related to cell accessibility, storage, and expansion [19].

Periodontal ligament stem cells (PDLSCs), located within periodontal ligaments, have emerged as critical progenitor cells in the processes of alveolar bone repair and regeneration [22–24]. Contemporary studies have demonstrated that the extensive capabilities of PDLSCs within regenerative medicine are significantly influenced by their secreted bioactive compounds, such as exosomes (small extracellular vesicles [sEVs]), cytokines, chemokines, and growth factors [25]. PDLSC-derived exosomes (PDLSC-Exos) represent a form of naturally occurring lipid nanosized vesicles (50 to 200 nm diameter) encapsulating bioactive elements, including functional proteins, microRNAs (miRNAs), and messenger RNAs (mRNAs), and can be transferred to target cells [26]. PDLSC-Exos transport genetic materials and transcription factors that play a vital role in cellular communication and mediate the cellular activities of recipient cells [27]. They exhibit essential biological roles, including the stimulation of cellular proliferation, the inhibition of apoptosis, the promotion of angiogenesis, and the enhancement of tissue repair capabilities [28], thereby presenting promising applications in the modulation of periodontal tissue regeneration.

Given the unique regenerative potential of PDLSCs, their exosomes hold potential for various therapeutic applications. To date, no review has been published assessing the role of PDLSC-Exos in tissue regeneration. Hence, this review explored the emerging role of PDLSC-Exos in both medicine and dentistry, particularly focusing on their regenerative potential for the treatment of periodontitis.

## Methods

An initial search using terms associated with exosomes (i.e., exosomes, exosomal, extracellular vesicles, and EVs) and periodontal ligament stem cells without any limitation of publication year and field of study was performed on October 31 st, 2024. A total of 638 publications were retrieved, including Elsevier’s Scopus (*n* = 302), Clarivate Analytics’ Web of Science (*n* = 179), and PubMed/MEDLINE (*n* = 157) (Table S1). Of these, 497 were selected and further assessed. Following the full-text screening, a total of 44 research articles were included. The included studies examined the therapeutic potential of PDLSC-Exos for the treatment of medical and dental tissues/diseases.

## Results

### Primary features of in-vitro studies

Table S2 presents the primary features of the included *in-vitro* studies. The majority of the studies were conducted in China, followed by Australia, France, Iraq, Iran, Italy, Japan, Korea, and Turkey. The predominant research focused on the use of PDLSC-Exos for the treatment of periodontitis; while additional studies explored their use for the management of bone defects, dental caries, diabetes mellitus, dry eye disease, inflammatory bowel disease (IBD), multiple sclerosis, oncogenesis, orthodontic tooth movement (OTM), and orthodontically-induced inflammatory root resorption. Primary cell sources were premolars, third molars, and PDLSCs, ranging from passages P2-P10. A few studies used donated or company-provided cells. Culture media primarily included the Dulbecco’s modified eagle medium (DMEM) or minimum essential medium eagle-alpha modification (α-MEM) supplemented with 10–20% fetal bovine serum (FBS), antibiotics (penicillin/streptomycin), and sometimes additional supplements such as L-glutamine, ascorbic acid, β-glycerophosphate, and dexamethasone for osteogenic differentiation.

Differential ultracentrifugation was the most common method utilized for exosome isolation; other methods included polymer-based precipitation, ultrafiltration, and size-exclusion chromatography. The concentration of exosomes used varied substantially, ranging from as low as 0.05 µg/mL [29] to as high as 5000 µg/mL [30], with commonly utilized doses including 1, 5, 10, 50, 100, 200, and 500 µg/mL. A few studies reported broader dose–response doses (i.e., 25–500 µg/mL or 2–1000 µg/mL), while one study reported concentrations in particles/mL (i.e., 10^10^ particles/mL) [31]. The most frequently measured markers included transcription factors (runt-related transcription factor 2 [RUNX2] and osterix [OSX]), osteogenic proteins (osteocalcin [OCN], collagen type I alpha 1 [COL1A1], and alkaline phosphatase [ALP]), pathways-related markers (β-catenin, wingless-type [WNT] proteins, and SMADs), inflammatory markers (interleukin [IL]−6, tumor necrosis factor-alpha [TNF-α], IL-10), and miRNAs (miR-34c-5p, miR-205-5p).

A broad range of techniques were used to assess cellular responses such cell viability and proliferation assays (cell counting kit-8 [CCK-8], 3-(4,5-dimethylthiazol-2-yl)−2,5-diphenyltetrazolium bromide [MTT], 3-(4,5-dimethylthiazol-2-yl)−5-(3-carboxymethoxyphenyl)−2-(4-sulfophenyl)−2H-tetrazolium [MTS], 2,3-bis-(2-methoxy-4-nitro-5-sulfophenyl)−2H-tetrazolium-5-carboxanilide [XTT], 5-ethynyl-2'-deoxyuridine [Edu cell proliferation assay], colony forming unit [CFU], seahorse metabolic flux assay, and lactate and ATP assay), cell migration (cell scratch assay), cell staining and imaging (alizarin red S [ARS] and alkaline phosphatase staining, PKH26 and PKH67 staining, and immunofluorescence), morphological characterization (transmission [TEM] and scanning electron microscopy [SEM], atomic force microscopy [AFM], and cryo-electron microscopy), particle characterization (nanoparticle tracking analysis [NTA], dynamic light scattering [DLS], and flow cytometry), protein analysis (western blotting and enzyme-linked immunosorbent assay [ELISA]), gene expression (quantitative real-time polymerase chain reaction [qRT-PCR] and next-generation sequencing [NGS]), reporter assay (dual-luciferase reporter assay), spectroscopy (Fourier-transform infrared spectroscopy [FTIR]), chromatography (high-performance liquid chromatography [HPLC]), and mass spectrometry (liquid chromatography-tandem mass spectrometry [LC–MS/MS]).

### Primary features of in-vivo studies

Table S3 presents the primary features of the included *in-vivo* studies. For the experimental modes, the predominantly used species were Sprague–Dawley rats (weight ranging from 103–350 g), C57BL/6 J mice (20–25 g), and Wistar rats (300–500 g). The ages ranged between 6 weeks and 24 months, and the study primarily used male animals. Sample sizes varied significantly across studies, ranging from 6 to 40 animals. Common research groups included (1) Control groups: untreated, PBS-treated, or defect-only groups; (2) Exosomes-treated groups: PDLSC-Exos or other functionalized exosome treatments; and (3) Comparative groups: autografts, hydrogels, or scaffolds without exosomes, and alternative treatments such as aspirin, miRNA-overexpressed exosomes, or encapsulated drugs. Exosome concentration demonstrated wide variability, ranging from as low as 1 μg/mL [32] to as high as 150,000 μg/mL [33], with several commonly used concentrations being 50, 100, 500, 1000, 2000, and 4000 μg/mL, while two studies administered a fixed dose per animal (i.e., 24 μg/mouse) [34, 35]. Frequently used assays to evaluate outcomes included imaging and structural analysis (micro-computed tomography [μ-CT], Masson’s staining, and hematoxylin and eosin [H&E] staining to assess bone formation), molecular analysis (qRT-PCR, western blotting, ELISA, and immunohistochemistry to detect biomarker expression), and functional analysis (immunofluorescence, tartrate-resistant acid phosphatase [TRAP] staining, and dual luciferase reported assays to analyze inflammation, osteogenesis, and macrophage polarization).

### How PDLSC-exos promotes periodontal regeneration?

PDLSCs secrete exosomes via a paracrine mechanism to facilitate intercellular communication, activate signaling pathways in target cells, and influence the biological functions of cells within a particular microenvironment, ultimately promoting the repair and regeneration of injured periodontal tissues [36]. While the precise mechanisms by which PDLSC-Exos contribute to periodontal regeneration are yet to be completely elucidated, numerous investigations have been conducted recently to explore the fundamental processes involved, which will be detailed in the following sections.

#### Enhanced proliferation and osteoblast differentiation

PDLSC-Exos promote cell proliferation and osteoblast differentiation through several mechanisms: (1) miRNA regulation; and (2) activation of insulin, AMPK, MPAK, Wnt/β-catenin, and miR-181b-5p/PTEN/AKT signaling pathways [37, 38].

##### Periodontitis

Lei et al. [33] demonstrated that healthy human PDLSCs (hPDLSCs)-Exos enhanced the osteodifferentiation of inflammatory PDLSCc (iPDLSCs), resulting in increased mineralized nodule formation and expression of osteogenic proteins and genes. Wang et al. [39] showed that tension-stimulated PDLSC-Exos, functionalized with miR-200b/c, promoted osteodifferentiation through the BMP-Smad signaling pathway and enhanced osteogenesis *in-vivo*. Lan et al. [38] showed that curcumin-primed PDLSC-Exos promoted osteoblast proliferation, migration, and osteodifferentiation via activating the Wnt/β-catenin signaling pathway, offering a potential solution for the treatment of periodontitis. Niu et al. [40] demonstrated that PDLSC-Exos overexpressing FoxO1 enhanced osteogenesis, immunomodulation and promoted osteoblast differentiation of PDLSCs. Lu et al. [41] found that PDLSC-Exos modulated alveolar bone regeneration in diabetic mice with periodontitis by delivering miR-31-5p, which targets eNOS to suppress osteoclast differentiation and decrease bone destruction *in-vivo*, with exosomes derived from normal glucose-cultured PDLSCs being more efficacious as compared to those from high-glucose-preconditioned PDLSCs. Dai et al. [42] demonstrated that gallic acid (GA) induction promoted the proliferation and osteodifferentiation of inflammatory PDLSCs via a decrease in oxidative stress and by enhancing mitochondrial function and glucose metabolism. Moreover, GA-induced PDLSC-Exos significantly enhanced osteoblast differentiation, offering a potential strategy to treat inflammatory bone loss in periodontitis. Novello et al. [43] found that exosomes present in the conditioned medium from PDLSCs facilitated osteoblast proliferation and the promotion of osteoblastic differentiation markers in Saos-2 cells. The impacts noticed appeared to be owing to the cumulative action of several constituents, with exosomes playing a vital part in modulating these therapeutic effects [43]. Lu et al. [44] showed that PDLSCs enhanced osteogenesis in BMSCs via the release of exosomes, with the process mediated by Rab27a. PDLSC-Exos were found to promote BMSCs osteodifferentiation *in-vitro* and enhanced bone regeneration *in-vivo*, with BMSCs rapidly internalizing PDLSC-Exos through the lipid raft/cholesterol pathway, activating ERK1/2 phosphorylation. These outcomes highlight the potential of PDLSC-Exos as a cell-free strategy for bone regeneration.

Yu et al. [45] revealed that PDLSC-Exos cultured in a three-dimensional (3D) strain microenvironment demonstrated altered properties and improved bioactivity, including variations in miRNA surface protein expression, size distribution, morphology, and content. These exosomes significantly enhanced the proliferation, migration, and osteodifferentiation of target cells, including BMSCs. It was further revealed that these exosomes enhanced greater alveolar bone regeneration *in-vivo* in experimental rats in comparison with exosomes from non-strained 3D culture, indicating that engineered mechanical microenvironments could be utilized to further promote exosome-based treatments in regenerative medicine. Lan et al. [46] investigated the potential of PDLSC-Exos in osteoblasts. It was concluded that PDLSC-Exos promoted the proliferation, migration, and osteodifferentiation of human osteoblast-like cells (hFOB1.19), while also suppressing hydrogen peroxide-induced apoptosis. The therapeutic effects were associated with the triggering of the MEK/ERK and PI3K/AKT signaling pathways [46].

##### Orthodontic tooth movement

Liu et al. [47] found that exosomal simvastatin promoted osteogenesis by increasing the expression of osteogenic-associated proteins and genes, thereby alleviating bone-resorptive lacunae, hence enhancing bone stability. Huang et al. [48] demonstrated that mechanical force promoted exosome biogenesis from PDLSCs via elevating the levels of annexin A3 (ANXA3), which enhanced exosome internalization and activated ERK signaling to induce osteoclast differentiation, hence affecting bone metabolism. Xu et al. [32] demonstrated that PDLSC-Exos promoted tension-induced osteogenesis by enhancing osteoblast proliferation and differentiation under tensile strain *in-vitro*. *In-vivo*, PDLSC-Exos enhanced OTM and osteogenesis in the tension group via increased trabecular bone formation and the expression of OPN and OCN. High-throughput miRNA sequencing revealed mechano-sensitive miRNAs in exosomes that contributed towards osteogenesis-associated signaling cascades [32].

##### Orthodontically-induced inflammatory root resorption

Li et al. [49] explored the potential of PDLSC-Exos in promoting the biological function of cementoblasts, crucial cells in the formation of cementum. PDLSs-Exos enhanced the proliferation, migration, and cementogenic mineralization of OCCM-30 cementoblasts, significantly increasing the expression of cementogenesis-associated proteins and genes. The effects were modulated via the PI3K/AKT signaling cascade. Inhibiting this pathway with a PI3K/AKT inhibitor decreased the effects, which were partially reversed by PDLSC-Exos [49]. These results indicate PDLSC-Exos are a promising therapeutic approach to enhance cementum repair in conditions such as orthodontically-induced inflammatory root resorption.

##### Bone regeneration

**Fig. 1:**
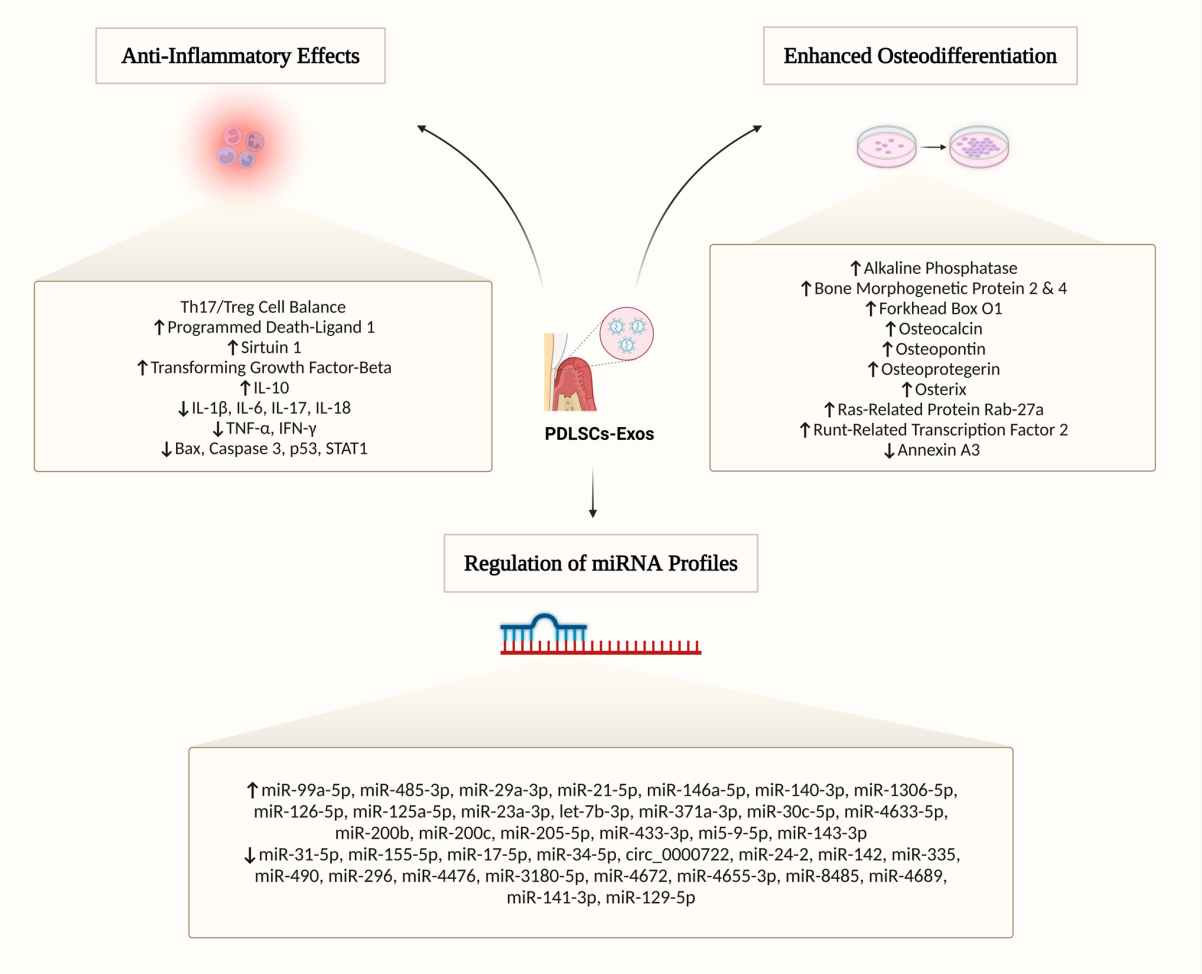
The role of periodontal ligament stem cells-derived exosomes via anti-inflammatory effects (Top left panel), enhanced osteogenic differentiation (Top right panel), and modulation of miRNA profiles (bottom panel)

Isik et al. [50] reported that hPDLFs-Exos, integrated into GelMA hydrogels, upregulated OSP, ALP, and RUNX2, thereby promoting osteogenesis and bone regeneration. Wang et al. [51] revealed that PDLSC-Exos cultured at an initial density of 2 × 10^4^ cells/cm^2^ significantly promoted osteodifferentiation of hBMSCs. The exosomes enhanced the highest gene expression of osterix (SP7), RUNX2, OCN, and osteoprotegerin (OPG), as well as increased levels of calcium and the OPG/RANKL (receptor activator of nuclear factor kappa beta ligand) ratio, highlighting the potential role of PDLSC-Exos in modulating osteogenesis. A study by Han et al. [52] investigated the impacts of different exosomal subtypes derived from various periodontal cells, i.e., gingival fibroblasts, osteoblasts, and PDLSCs, on buccal fat pad-derived MSCs. The findings revealed that PDLSC-Exos enhanced proliferation, migration, and osteodifferentiation, with osteoblasts-Exos demonstrating superior levels of osteogenic markers. Albougha et al. [53] demonstrated that PDLSC-Exos enhanced osteogenesis in both *in-vitro* and *in-vivo* models. PDLSC-Exos promoted the migration, mineralization, and expression of OPN, OCN, BMP2, and ALP in human osteoblast-like cells. *In-vivo*, PDLSC-Exos enhanced bone regeneration in rat calvarial defects, indicating that exosomes might be a promising therapeutic approach to healing injured periodontal tissues. Table 1 and Fig. 1 (top right panel) represent the role of PDLSC-Exos in bone regeneration via enhancing osteogenic differentiation.

**Table 1:** Summary of studies focused on the role of PDLSC-Exos in bone regeneration via enhancing osteogenic differentiation

Study	Source	Target	Mechanism	miRNAs/Proteins	Outcome
Periodontitis					
Lei et al. [28]	hPDLSC-Exos	iPDLSCs	N/A	Osteogenic proteins and genes	**↑**Osteodifferentiation **↑**Mineralized nodule formation
Wang et al. [29]	Tension-stimulated PDLSC-Exos	hBMSCs	BMP-Smad signaling	miR-200b/c	**↑**Osteodifferentiation **↑**Osteogenesis *in-vivo*
Lan et al. [27]	Curcumin-primed PDLSC-Exos	Osteoblasts	Wnt/β-catenin signaling	N/A	**↑**Proliferation, migration, and osteodifferentiation of osteoblasts
Niu et al. [30]	PDLSC-Exos	PDLSCs	N/A	FoxO1	**↑**Osteogenesis **↑**Immunomodulation
Lu et al. [31]	PDLSC-Exos (miR-31-5p)	N/A	eNOS signaling	miR-31-5p	**↓**Osteoclast differentiation **↓**Bone destruction in diabetics
Dai et al. [32]	GA-induced PDLSC-Exos	iPDLSCs	Mitochondrial function and glucose metabolism	N/A	**↑**Osteodifferentiation **↓**Oxidative stress
Novello et al. [33]	PDLSC-Exos	Saos-2 cells	N/A	Osteoblastic differentiation markers	**↑**Proliferation and osteoblast differentiation
Lu et al. [34]	PDLSC-Exos	BMSCs	Lipid raft/cholesterol pathway, ERK1/2 phosphorylation	Rab27a	**↑**Osteogenesis *in-vitro* and *in-vivo*
Yu et al. [35]	PDLSC-Exos	BMSCs	N/A	N/A	**↑**Bioactivity, proliferation, and osteodifferentiation under a 3D strain microenvironment
Lan et al. [36]	PDLSC-Exos	hFOB1.19 cells	MEK/ERK and PI3K/AKT pathways	N/A	**↑**Proliferation, migration, and osteodifferentiation
Orthodontic tooth movement					
Lui et al. [37]	Exosomal simvastatin	N/A	N/A	Osteogenic-associated proteins and genes	**↑**Osteogenesis, **↓**Bone-resorptive lacunae **↑**Improved bone stability
Huang et al. [38]	PDLSC-Exos	Osteoclasts	ERK signaling	Annexin A3	**↑**Exos biogenesis **↑**Osteoclast differentiation
Xu et al. [39]	PDLSC-Exos	Osteoblasts	N/A	OPN, OCN	**↑**Tension-induced osteogenesis **↑**Orthodontic tooth movement
Orthodontically-induced inflammatory root resorption					
Li et al. [40]	PDLSC-Exos	OCCM-30 cementoblasts	PI3K/AKT signaling	Cementogenesis-associated proteins and genes	**↑**Proliferation, migration, and cementogenic mineralization
Other bone defects					
Isik et al. [41]	hPDLF-Exos	N/A	N/A	OSP, ALP, RUNX2	**↑**Markers of osteogenesis **↑**Bone regeneration
Wang et al. [42]	PDLSC-Exos	HBMSCs	N/A	RUNX2, SP7, OPG, OCN	**↑**Gene expression **↑**Calcium levels Modulated osteogenesis
Han et al. [43]	PDLSC-Exos	PDLSCs	N/A	N/A	**↑**Proliferation, migration, and osteodifferentiation of PDLSCs
Albougha et al. [44]	PDLSC-Exos	Osteoblast-like cells	MEK/ERK and PI3K/AKT pathways	OPN, OCN, BMP2, ALP	**↑**Migration, mineralization, and expression of osteogenic markers

#### Anti-inflammatory effects

PDLSC-Exos exhibit anti-inflammatory characteristics that assist in alleviating the inflammatory environment that is prevalent in periodontitis. Exosomes have been shown to modulate the expression of pro-inflammatory cytokines such as interleukin (IL)−1β, IL-6, and tumor necrosis factor-alpha (TNF-α), by influencing signaling pathways such as the IL-6/JAK2/STAT3 and Th17/Treg/miR‐155‐5p/SIRT1 pathways [33, 54]. This modulation creates an environment conducive to tissue regeneration.

##### Periodontitis

Shi et al. [55] demonstrated that aspirin-loaded PDLSC-Exos decreased inflammation in macrophages, enhanced anti-inflammatory macrophage polarization, and improved oxidative phosphorylation. In the periodontitis model, these exosomes enhanced the immune microenvironment, alleviated bone loss, and facilitated alveolar bone regeneration. Kang et al. [56] demonstrated that exosomal miR-205-5p derived from PDLSCs facilitated a reduction in inflammation in periodontitis via targeting XBP1. In LPS-treated cells and rats, these exosomes alleviated inflammatory cytokines (IL-6, IL-1β, TNF-α), Th17 cells, and immune cell proliferation. Their anti-inflammatory impacts were associated with the regulation of Th17/Treg cell balance [56]. Zheng et al. [57] investigated the anti-inflammatory effects of PDLSC-Exos in chronic periodontitis. They found that exosomes from LPS-stimulated PDLSCs facilitated restoring the Th17/Treg cell balance, which is altered in periodontitis. These exosomes exhibited higher SIRT1 and lower miR-155-5p levels, affecting the inflammatory environment.

##### Dental caries

Pourhajibagher et al. [58] assessed the application of emodin-loaded PDLSC-Exos (Emo@PDLSC-Exos) in antimicrobial photodynamic therapy (aPDT) against *Lactobacillus acidophilus* and *Streptococcus mutans*. They demonstrated that Emo@PDLSC-Exos efficiently decreased metabolic activity, biofilm formation, and bacterial viability. It also alleviated the expression of virulence genes (*slpA* and *gtfB*) owing to an increased formation of ROS.

##### Inflammatory bowel disease

Tang et al. [59] reported that thermally engineered PDLSC-Exos improved the upregulation of PD-L1 expression, significantly enhancing their anti-inflammatory efficacy. These exosomes efficiently mitigated symptoms and pathological damage in a murine colitis model by mediating the Th17/Treg cell equilibrium.

##### Multiple sclerosis

Rajan et al. [34] revealed that PDLSC-Exos and PDLSC conditioned medium exerted anti-inflammatory effects in a mouse model of multiple sclerosis. Treatment with these PDLSCs-derived products decreased inflammation via suppressing the NALP3 inflammasome, alleviating the levels of pro-inflammatory cytokines (IL-18 and IL-1β), and inhibiting the NF-κB pathway. Moreover, these treatments increased a number of anti-inflammatory cytokines such as TGF-β and IL-10, facilitating a more balanced immune response. In another study conducted by the same research group [35], they reported that PDLSC-Exos and conditioned medium exerted immunosuppressive and anti-inflammatory effects in various mouse models with multiple sclerosis. These treatments decreased pro-inflammatory cytokines (TNF-α, IL-6, IL-1β, IFN-γ, and IL-17) and apoptosis-associated proteins (Bax, Caspase 3, p53, and STAT1), while also increasing the anti-inflammatory cytokine IL-10. These treatments were shown to facilitate reversing disease advancement and enhance tissue repair. Table 2 and Fig. 1 (top left panel) represent the role of PDLSC-Exos in bone regeneration via anti-inflammatory effects.

**Table 2:** Summary of studies focused on the role of PDLSC-Exos in bone regeneration via anti-inflammatory effects

Study	Source	Target	Mechanism	miRNAs/Proteins	Outcome
Periodontitis					
Shi et al. [46]	Aspirin-loaded PDLSC-Exos	Macrophages	Anti-inflammatory, macrophage polarization, oxidative phosphorylation	N/A	**↓**Inflammation **↑**Immune microenvironment **↓**Bone loss **↑**Alveolar bone regeneration
Kang et al. [47]	PDLSC-Exos (miR-205-5p)	XBP1, Th17/Treg cells	miR-205-5p targeting XBP1, regulating Th17/Treg balance	miR-205-5p, IL-6, IL-1β, TNF-α	**↓**Inflammation in periodontitis by regulating cytokines and immune cell proliferation
Zheng et al. [48]	LPS-stimulated PDLSC-Exos	Th17/Treg cells, SIRT1	Restored Th17/Treg balance, upregulated SIRT1, downregulated miR-155-5p	SIRT1, miR-155-5p	Restored Th17/Treg cell balance **↓**Inflammatory environment in chronic periodontitis
Dental caries					
Pourhajibagher et al. [49]	Emodin-loaded PDLSC-Exos	*L. acidophilus, S. mutans*	Reduced bacterial viability, biofilm formation, ROS production	N/A	**↓**Expression of virulence genes **↓**Bacterial metabolic activity in aPDT
Inflammatory bowel disease					
Tang et al. [50]	Thermally engineered PDLSC-Exos	PD-L1, Th17/Treg cells	PD-L1 upregulation, Th17/Treg balance	PD-L1, Th17/Treg balance	**↑**Anti-inflammatory efficacy **↓**Symptoms and damage in murine colitis model
Multiple sclerosis					
Rajan et al. [51]	PDLSC-Exos and conditioned medium	NALP3 inflammasome, cytokines	Suppressed NALP3 inflammasome, inhibited NF-κB, upregulated TGF-β and IL-10	TGF-β, IL-10, IL-18, IL-1β	**↓**Inflammation in multiple sclerosis model Balanced immune response **↑**Tissue repair
Rajan et al. [52]	PDLSC-Exos and conditioned medium	Cytokines, apoptosis-associated proteins	Suppressed pro-inflammatory cytokines and apoptosis	TNF-α, IL-6, IL-1β, Bax, Caspase 3, p53, STAT1	**↓**Inflammation **↑**Tissue repair **↑**Immune modulation in MS model

##### Regulation of microRNA profiles

miRNAs are small non-coding RNAs, approximately 20–24 nucleotides in length. Within the interaction network of miRNA and mRNA, a single miRNA can bind with multiple mRNAs, thereby mediating several signaling pathways [60]. Research has indicated that miRNAs are significantly abundant in exosomes [61]. The presence of miRNAs within exosomes serves as a critical element for intercellular communication [62] and exhibits variability depending on the culture conditions used [63]. Specifically, miR-146a and miR-155 are secreted by activated dendritic cells in exosomes and subsequently internalized by recipient dendritic cells from the bone marrow [64]. Exosomes possess the capability to modulate epigenetic mechanisms by transporting miRNAs to target cells, thereby influencing the biological activities of recipient cells during the process of bone regeneration [65, 66]. Nonetheless, the function of PDLSC-Exos in the context of bone regeneration, as well as their specific miRNA profiles, has yet to be elucidated.

##### Periodontitis

Lin et al. [29] explored how exosomes containing miR-34c-5p from PGE2-induced PDLFs influenced the osteogenic role of PDLSCs. The exosomal miR-34c-5p suppressed osteogenesis in PDLSCs via decreasing ALP activity, mineralization, and ERK1/2 phosphorylation. It targeted SATB2, a crucial mediator, via the SATB2/ERK pathway. These outcomes indicate that inflammatory exosomes containing miR-34c-5p inhibit PDLSCs osteogenesis, providing insights into the molecular mechanisms of bone remodeling in inflammatory environments. Wang et al. [39] investigated the modulation of osteogenesis by exosomes derived from tension-stimulated PDLSCs. They found miR-200b/c as mechano-responsive miRNAs in PDLSC-Exos under tension. These miRNAs were taken up by human mandibular BMSCs, enhancing their osteogenesis by targeting Smurf1 and activating the BMP-Smad signaling cascade. *In-vivo*, miR-200b/c-functionalized exosomes were demonstrated to promote the repair of alveolar bone defects in a rat model, indicating their potential as a therapeutic tool for bone loss in periodontitis. Xie et al. [67] demonstrated that exosomes derived from osteogenically-induced PDLSCs enhanced osteoclastogenesis in RAW264.7 cells. These exosomes were enriched with circ_0000722, which promoted osteoclast formation via upregulating TRAF6 and activating AKT and NF-κB pathways. Downregulation of circ_0000722 decreased the osteoclast-enhancing impacts of these exosomes, highlighting its vital role in bone remodeling.

##### Orthodontic tooth movement

Zheng et al. [68] investigated the role of mechanical forces and their influence on miRNAs in PDLSC-Exos. They found that mechanical stress significantly changed the profile of miRNAs, with 10 miRNAs identified as differentially expressed including miR-99a-5p, miR-485-3P, miR-29a-3p,miR-21-5p, miR-146a-5p, miR140-3p, miR-1306-5p, miR-126-5p, miR-125a-5p, and miR-23a-3p. These miRNAs were associated with osteoclast and osteoblast activity, indicating a direct role in bone remodeling. Chang et al. [69] found that cyclic tension stretching of PDLSCs promoted the migration of BMSCs via exosomes. The miRNA profiles of exosomes from stretched PDLSCs exhibited upregulation of let-7b-3p, miR-371a-3p, miR-30c-5p, miR-4633-5p, and downregulation of miR-4476, miR-3180-5p, miR-4672, miR-4655-3p, miR-8485, and miR-4689. Bioinformatics analysis showed that the target genes of these miRNAs contributed to vesicle transmission and PI3K pathway, indicating a modulatory role in BMSCs migration during OTM.

##### Diabetes mellitus

Liu et al. [70] found that PDLSC-Exos counteract high-glucose-induced senescence in PDLSCs via delivering miR-141-3p, which inhibits KEAP1 and activates the NRF2 pathway. This alleviated oxidative stress and enhanced cellular rejuvenation, suggesting PDLSC-Exos as a promising approach for periodontal tissue regeneration. Zhong et al. [71] found that high-glucose disrupts endoplasmic reticulum (ER) calcium homeostasis in PDLSCs via increasing intracellular miR-129-3p, which downregulated TMCO1 and undermined osteogenesis through RUNX2 degradation. Metformin restored ER equilibrium by improving the secretion of exosomes, decreasing miR-129-3p levels, and rescuing osteogenic activity. A nanocarrier, Met@HALL, for local metformin delivery enhanced the remodeling of periodontal tissues in diabetic models.

##### Cancer

Chiricosta et al. [72] identified five important miRNAs (miR24-2, miR142, miR335, miR490, and miR296) in PDLSC-Exos. These miRNAs target genes were found to be involved in “cytoskeleton organization” and “Ras protein signal transduction”, and might also contribute to silencing proto-oncogenes linked with several tumors, indicating their potential effect on tumor modulation and cellular signaling.

##### Bone regeneration

Liu et al. [37] reported that osteogenic induction improved the osteogenic potential of PDLSC-Exos, enhancing BMSCs differentiation. RNA sequencing demonstrated 35 downregulated and 72 upregulated miRNAs in exosomes during osteodifferentiation of PDLSCs. These miRNAs target genes are involved in catalytic activity, protein binding, and differentiation, affecting signaling cascades such as insulin signaling, AMPK, and MAPK, which are all vital for osteogenesis. Table 3 and Fig. 1 (bottom panel) represent the role of PDLSC-Exos in bone regeneration via regulation of miRNA profiles.

**Table 3:** Summary of studies focused on the role of PDLSC-Exos in bone regeneration via regulation of miRNA profiles

Study	miRNAs identified	Mechanism	Outcome
Periodontitis			
Lin et al. [60]	miR-34c-5p	Exosomal miR-34c-5p from PGE2-induced PDLFs suppressed osteogenesis in PDLSCs via the SATB2/ERK pathway	Highlighted the inhibitory role of inflammatory Exos in bone remodeling under inflammatory conditions
Wang et al. [29]	miR-200b, miR-200c	Tension-stimulated PDLSC-Exos enriched with miR-200b/c enhanced osteogenesis in mandibular BMSCs via BMP-Smad signaling	miR-200b/c-functionalized Exos promoted alveolar bone defect repair in rat models
Xie et al. [61]	circ_0000722	Osteogenically induced PDLSC-Exos enriched with circ_0000722 enhanced osteoclastogenesis via TRAF6, AKT, and NF-κB pathways	Demonstrated circ_0000722’s role in bone remodeling by promoting osteoclast activity
Orthodontic tooth movement			
Zheng et al. [62]	miR-99a-5p, miR-485-3p, miR-29a-3p, miR-21-5p, miR-146a-5p, miR-140-3p, miR-1306-5p, miR-126-5p, miR-125a-5p, miR-23a-3p	Mechanical stress altered PDLSC-Exos miRNA profiles, including miRNAs linked to osteoclast and osteoblast activity	Identified miRNA changes as mediators of bone remodeling under mechanical stress
Chang et al. [63]	Upregulated: let-7b-3p, miR-371a-3p, miR-30c-5p, miR-4633-5p Downregulated: miR-4476, miR-3180-5p, miR-4672, miR-4655-3p, miR-8485, miR-4689	Cyclic tension in PDLSCs altered exosomal miRNA profiles, promoting BMSCs migration and influencing the PI3K pathway	Indicated the role of PDLSC-Exos in BMSC migration during OTM
Diabetes mellitus			
Liu et al. [64]	miR-141-3p	PDLSC-Exos delivered miR-141-3p, counteracting high-glucose-induced senescence by activating the NRF2 pathway	**↑**Oxidative stress response and cellular rejuvenation, suggesting potential for periodontal regeneration
Zhong et al. [65]	miR-129-3p	High glucose increased miR-129-3p, disrupting ER calcium balance and osteogenesis. Metformin and Exos restored balance	Nanocarrier Met@HALL enhanced periodontal tissue remodeling in diabetic models
Cancer			
Chiricosta et al. [66]	miR-24–2, miR-142, miR-335, miR-490, miR-296	PDLSC-Exos contained miRNAs targeting “cytoskeleton organization” and “Ras protein signal transduction”	Suggested roles in cellular signaling, tumor modulation, and proto-oncogene silencing
Other bone defects			
Liu et al. [26]	35 downregulated, 72 upregulated miRNAs (specific names not provided)	Osteogenic induction of PDLSC-Exos altered miRNA profiles, enhancing BMSCs differentiation and osteogenesis	Identified signaling cascades (e.g., insulin, AMPK, MAPK) vital for bone regeneration

#### Promotion of angiogenesis

Bone regeneration represents a process of considerable intricacy, fundamentally reliant on the interactions among diverse cellular types. Specifically, periodontal and osseous tissues are characterized by a significant vascular network, highlighting the profound interconnection between angiogenesis and tissue regeneration. Blood vessels serve as conduits for the transport of minerals, growth factors, and other essential components into a regenerative microenvironment [73]. Additionally, they function as a structural framework upon which the initiation of bone formation propagates. Moreover, blood vessels fulfill an additional role termed angiocrine function, via paracrine signaling, influencing the growth, differentiation, and regeneration of various cell types, including those involved in bone and periodontal tissues [74, 75].

The impact of vascularization is a critical consideration within regenerative medicine, which aims to develop biomaterials/growth factors to address deficiencies resulting from injuries or pathological conditions when natural tissue regeneration proves insufficient. In fact, following the *in-vivo* implantation of a bone graft, for instance, the establishment of vascular networks is essential for sustaining cellular viability, as inadequate blood perfusion leads to deficits in nutrients and oxygen, ultimately resulting in cell apoptosis [73]. The interplay between periodontal tissues and blood vessels occurs throughout physiological development and during the processes of tissue regeneration or fracture healing [76]. For these reasons, various cell types, such as osteoblasts, possess the capability to secrete pro-angiogenic factors, such as VEGF [77].

VEGFA is integral to angiogenesis and is equally crucial during bone development and regeneration. Specifically, VEGF stimulates both the migration and proliferation of endothelial cells while concurrently enhancing osteogenesis through the regulation of osteogenic growth factors [73].

The VEGF family comprises multiple members; however, VEGFA, commonly referred to as VEGF, was the first member identified and plays a vital role in angiogenesis [78]. In addition to its release from endothelial cells, VEGF is also secreted by various other cell types, such as osteoblasts [79]. VEGF binds to the extracellular domains of two tyrosine kinase receptors: VEGF receptor 1 (VEGFR1) and VEGF receptor 2 (VEGFR2) [80]. The signaling of VEGFA in blood endothelial cells appears to be predominantly modulated via the stimulation of VEGFR2, which is implicated in angiogenesis, as well as in the proliferation, differentiation, and migration of endothelial cells [81]. Considering the significant role that angiogenesis plays in bone physiology, a strategy that enhances both vascularization and bone regeneration might represent the optimal therapeutic approach to repair bone defects [80].

##### Periodontitis

Zhang et al. [82] studied angiogenesis in periodontitis, focusing on the role of PDLSCs and exosomes-modulated pathways. The results found increased expression of VEGF and vascular marker CD31 in PDL under an inflammatory environment. It was found that the inflammatory-stimulated PDLSCs upregulated VEGFA expression and improved angiogenesis in HUVECs via exosome release. Inhibiting exosome secretion reduced angiogenesis, confirming the potent role of exosomes during angiogenic modulation. VEGFA transfer through exosomes was a vital process, mediated by inflammation-inhibited miR-17-5p in PDLSCs. Overexpression of miR-17-5p suppressed pro-angiogenic effects. These findings highlight that periodontitis enhanced angiogenesis through exosome-modulated VEGFA transfer, providing insights into vascular alterations in periodontal disease.

##### Bone regeneration

**Fig. 2:**
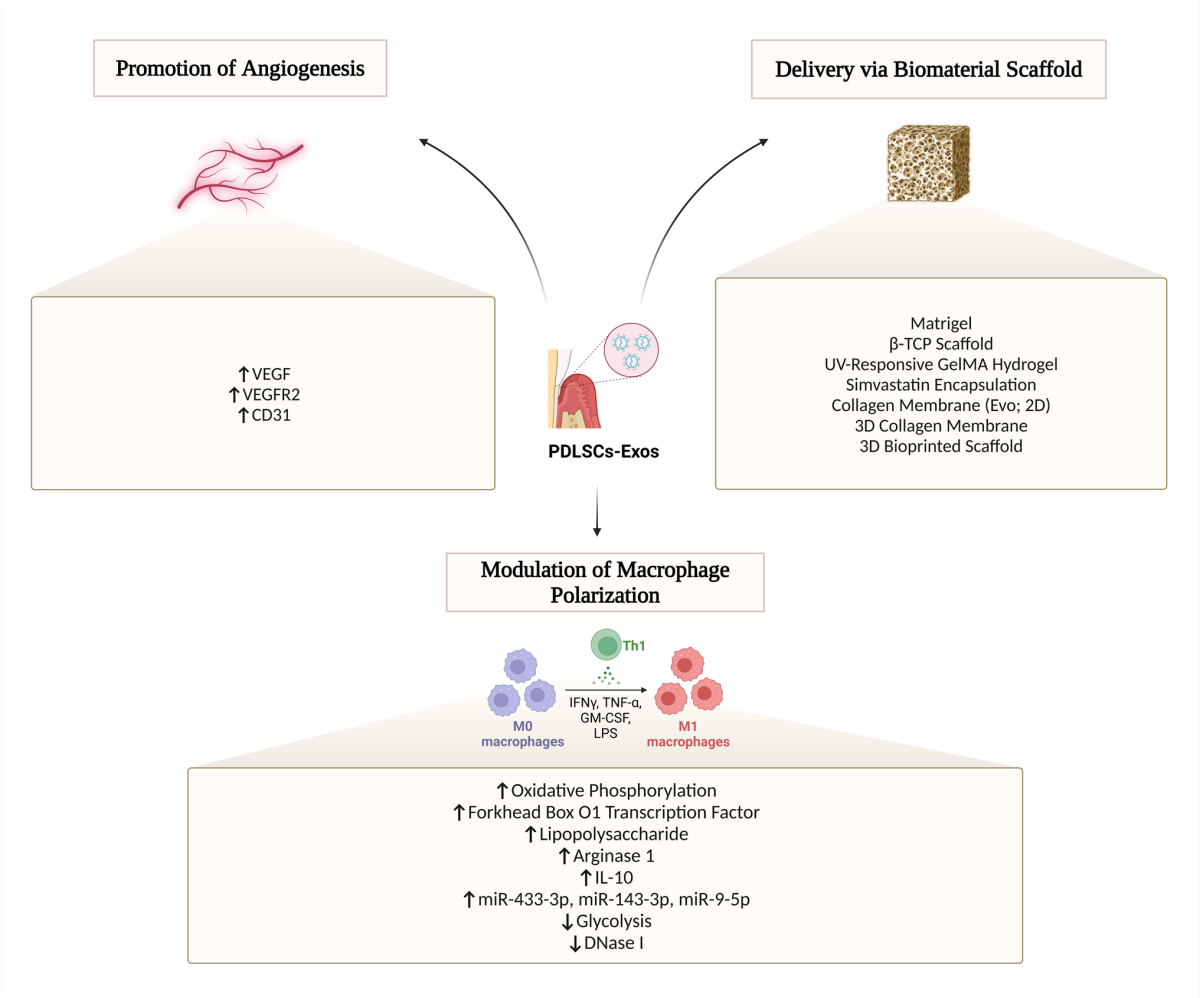
The role of periodontal ligament stem cells-derived exosomes via promotion of angiogenesis (Top left panel), delivery by biomaterial scaffolds (Top right panel), and modulation of macrophage polarization (bottom panel)

A study by Pizzicannella et al. [83] focused on the role of angiogenesis in bone regeneration utilizing a collagen membrane (3D-COL) enriched with PDLSCs and polyethylenimine-engineered exosomes (PEI-Exos). The combination significantly increased the expression of VEGF and VEGFR2 *in-vitro*, enhancing angiogenesis. *In-vivo*, rats implanted with the enriched 3D-COL demonstrated improved vascularization and bone regeneration in calvarial defects. These outcomes highlight the significance of enhancing vascularization together with osteogenesis for efficient bone repair. Table 4 and Fig. 2 (top left panel) represent the role of PDLSC-Exos via promotion of angiogenesis.

**Table 4:** Summary of studies focused on the role of PDLSC-Exos in bone regeneration via promotion of angiogenesis

Study	Source	Target	Mechanism	miRNAs/Proteins	Outcome
Periodontitis					
Zhang et al. [76]	PDLSC-Exos under inflammatory conditions	HUVECs	VEGFA transfer via exosomes, modulated by miR-17-5p	VEGFA, CD31, miR-17-5p	**↑**Angiogenesis **↓**miR-17-5p → **↑**VEGFA-mediated angiogenic effects
Other bone defects					
Pizzicannella et al. [77]	PEI-engineered exosomes (PEI-Exos) enriched with PDLSCs in 3D collagen membrane (3D-COL)	VEGF, VEGFR2	VEGF signaling	VEGF, VEGFR2	**↑**Angiogenesis *in-vitro* **↑**Vascularization and bone regeneration *in-vivo*

#### Modulation of macrophage polarization

In addition to strategies aimed at addressing periodontitis via the eradication of microbial biofilms, the concept of immunomodulation is garnering increasing interest among researchers as a potential adjunctive approach [84]. Macrophages, which constitute a pivotal component of the innate immune response, serve as central orchestrators in the inflammatory process [85], manifesting two distinct activation states: (a) the classically activated pro-inflammatory M1 phenotype; and (b) the alternative pro-resolution/anti-inflammatory M2 phenotype [86]. Previous investigations have indicated the capacity of macrophages for phenotypic plasticity, allowing for transitions between M1 and M2 polarization [87, 88]. Moreover, within the context of periodontal inflammation, macrophages can undergo a transformation from the M2 to the M1 phenotype [89, 90], leading to the secretion of significant levels of pro-inflammatory mediators and facilitating osteoclast activation [91], thereby contributing to the exacerbation of periodontitis. Consequently, a therapeutic approach aimed at modulating macrophage polarization might represent a promising avenue to treat periodontitis.

Exosomes have recently been recognized as potential agents for cell-to-cell communication and mediators of immunoregulatory processes [92]. Specifically, miRNAs function as regulatory molecules that can silence gene expression at the post-translational stage. Additionally, MSCs have been demonstrated to influence macrophage activity via exosomal miRNAs [93, 94]. Nevertheless, the specific mechanisms through which PDLSC-Exos impact macrophage polarization within an inflammatory periodontal microenvironment remain to be elucidated.

##### Periodontitis

Shi et al. [55] found that aspirin-loaded PDLSC-Exos mediate macrophage polarization via inhibiting glycolysis and promoting oxidative phosphorylation, shifting macrophages to an anti-inflammatory (M2-like) phenotype. This immune modulation enhanced the periodontal microenvironment, decreased bone loss, and facilitated alveolar bone regeneration in a periodontitis model, presenting a promising therapeutic strategy for bone repair. Niu et al. [40] reported that FoxO1 overexpressed PDLSC-Exos enhance M2 macrophage polarization, improved mitochondrial activity, mediated ROS levels, and facilitated osteogenesis. They alleviated inflammation and stimulated bone regeneration in periodontitis. A study by Kang et al. [95] highlighted the role of PDLSCs in macrophage modulation during inflammation. PDLSCs-preconditioned with lipopolysaccharide (LPS) significantly induced M1 macrophage polarization via exosomes. Notably, this M1 polarization effect was eliminated by DNase-I treatment of the exosomes, implicating DNA content within exosomes as a mediator. These outcomes indicated that exosomes from LPS-stimulated PDLSCs play a vital role in macrophage polarization and could serve as a potential therapeutic target for treating periodontal inflammation. Cui et al. [96] demonstrated that LPS-preconditioned PDLSC-Exos drive macrophage polarization towards the pro-inflammatory M1 phenotype. These exosomes increased inflammatory cytokines and M1 markers while decreasing anti-inflammatory cytokines and M2 markers. Mechanistically, the TLR2/TLR4/NF-κB p65 signaling cascade was identified as a key mediator in this polarization process, with miRNA-433-3p playing a contributory role. Wang et al. [84] reported that PDLSC-Exos enhanced pro-inflammatory M1 macrophage polarization via miR-143-3p. This miRNA, enriched in iPDLSC-Exos, inhibited the PI3K/AKT signaling pathway and stimulated NF-κB signaling, favoring M1 polarization. Inhibiting exosome generation or targeting the PI3K pathway decreased this effect. *In-vivo*, exosomal miR-143-3p increased periodontal inflammation, underscoring their role in immunomodulation and as a potential therapeutic target for periodontitis treatment. Wu et al. [97] demonstrated that mechanical stress-activated PDLSCs to release exosomes enriched with miR-9-5p, which promoted M1 macrophage polarization via the SIRT1/NF-κB signaling pathway. This response amplified periodontal inflammation, offering insights into force-associated inflammatory diseases and identifying potential therapeutic targets.

##### Dry eye disease

Ren et al. [98] demonstrated that PDSCs-Exos enhances the polarization of pro-inflammatory M1 macrophages into anti-inflammatory M2 macrophages, increasing Arg1 and IL-10 levels. This modulation protects conjunctival goblet cells and decreases inflammation via promoting Muc5ac expression, which is vital for mucosal health. Table 5 and Fig. 2 (bottom panel) represent the role of PDLSC-Exos via modulation of macrophage polarization.

**Table 5:** Summary of studies focused on the role of PDLSC-Exos in bone regeneration via modulation of macrophage polarization

Study	Macrophage polarization	Mechanism	Outcome
Periodontitis			
Shi et al. [46]	M2-like (Anti-inflammatory)	Glycolysis inhibition and oxidative phosphorylation promotion via aspirin-loaded PDLSC-Exos	**↑**Periodontal microenvironment **↓**Bone loss **↑**Alveolar bone regeneration
Nui et al. [30]	M2 (Anti-inflammatory)	FoxO1-overexpressed PDLSC-Exos improving mitochondrial activity, mediating ROS levels, and facilitating osteogenesis	**↓**Inflammation **↑**Bone regeneration
Kang et al. [89]	M1 (Pro-inflammatory)	DNA content in Exos from LPS-preconditioned PDLSCs inducing M1 polarization	Highlighted therapeutic potential of Exos for periodontal inflammation; effect nullified by DNase-I
Cui et al. [90]	M1 (Pro-inflammatory)	TLR2/TLR4/NF-κB p65 signaling cascade mediated by miRNA-433-3p from LPS-preconditioned PDLSC-Exos	**↑**Inflammatory cytokines and M1 markers **↓**Anti-inflammatory cytokines and M2 markers
Wang et al. [78]	M1 (Pro-inflammatory)	miR-143-3p in inflammatory PDLSC-Exos inhibiting PI3K/AKT and stimulating NF-κB signaling	**↑**Periodontal inflammation, highlighting miR-143-3p as a therapeutic target for periodontitis treatment
Wu et al. [91]	M1 (Pro-inflammatory)	miR-9-5p-enriched Exos from mechanically stressed PDLSCs activating SIRT1/NF-κB signaling	**↑**Periodontal inflammation, offering insights into force-associated inflammatory diseases
Dry eye disease			
Ren et al. [92]	M1 to M2 (Anti-inflammatory)	PDLSC-Exos promoting Arg1 and IL-10 levels to enhance M2 polarization	Protected conjunctival goblet cells **↓**Inflammation **↑**Mucosal health via Muc5ac expression

#### Delivery of exosomes via biomaterial scaffolds

Despite the numerous advantages associated with the utilization of exosomes, challenges persist regarding the administration of therapeutically effective doses. Exosomes can be readily eliminated from systemic circulation and might also preferentially accumulate in organs such as the lungs, spleen, liver, and gastrointestinal system [99]. Consequently, the effective delivery of exosomes necessitates the development of enhanced strategies to mitigate these concerns [100]. Presently, the demand for biomaterials that are biodegradable, bioactive, and biocompatible for exosome delivery has garnered significant interest within the realm of biomedical science [101–104].

The therapeutic potential of PDLSC-Exos can be amplified by integrating them into biomaterial scaffolds, which facilitate the gradual release of exosomes at the site of injury [105–107]. This strategy optimizes the benefits of exosome therapy in promoting the regeneration of periodontal tissues, including alveolar bone, periodontal ligament, and cementum.

##### Periodontitis

According to Lei et al. [33], when delivered via Matrigel or β-TCP scaffolds, PDLSC-Exos significantly enhanced bone regeneration in a rat model of periodontitis, exhibiting their potential as a cell-free therapy to repair inflammatory bone loss. Zhao et al. [19] further assessed the application of PDLSC-Exos immobilized in Matrigel for bone defect repair. In a rat calvarial defect model, Matrigel/PDLSC-Exos enhanced faster bone repair as compared to the control, and improved cell migration/infiltration. Han et al. [31] investigated the application of 3D bioprinted scaffolds incorporating PDLSC-Exos (GelMA/PDLCS-Exos) on tissue regeneration. The findings revealed that scaffolds made with PDLSC-Exos enhanced better attachment and differentiation of MSCs into periodontal ligament, bone, and cementum, when compared with scaffolds loaded with exosomes derived from gingival fibroblasts versus without exosomes.

##### Orthodontic tooth movement

Liu et al. [47] demonstrated that encapsulating simvastatin into PDLSC-Exos improved drug solubility and efficacy in decreasing relapse after OTM. In a rat model, local injection of exosomal simvastatin significantly suppressed relapse via an increase in osteogenesis, as evidenced by improved expression of osteogenic markers and reduced bone-resorptive lacunae. Importantly, PDLSC-Exos alone exhibited an ability to block relapse.

##### Bone regeneration

Isik et al. [50] reported that PDLFs-Exos integrated with UV-responsive GelMA hydrogels demonstrated significant potential for cell-free bone regeneration. The GelMA/PDLFs-Exos platform enhanced osteoblast differentiation in MSCs, as evidenced by the upregulation of OSP, ALP, and RUNX2. *In-vivo* studies in a rat calvarial defect model further revealed improved bone mineralization and regeneration, highlighting the hydrogel/Exo’s ability to recruit and instruct endogenous stem cells for bone repair. Diomede et al. [30] formulated a biocompatible osteogenic construct by combining a collagen membrane (Evo) with PDLSCs and exosomes, including polyethylenimine-engineered exosomes (PEI-Exos). *In-vitro* and *in-vivo* experiments showed that Evo enriched with exosomes and PEI-Exos demonstrated high biocompatibility and osteogenic potential. PEI-Exos improved the expression of BMP2 and BMP4, critical for bone regeneration. Furthermore, constructs using Evo, PDLSCs, and PEI-Exos significantly enhanced bone healing in rat calvarial defect models, exhibiting potential for repairing bone defects resulting from surgery or trauma [30]. Pizzicannella et al. [83] investigated the application of a 3D collagen membrane (3D-COL) enriched with PDLSCs and PEI-Exos for bone regeneration in calvaria defects. *In-vitro*, the scaffolds enhanced osteogenic expression and angiogenic proteins such as VEGF. *In-vivo*, rats treated with 3D-COL, PDLSCs, and PEI-Exos demonstrated improved bone regeneration and vascularization. Zhao et al. [108] evaluated the utility of PDLSC-Exos for bone regeneration. *In-vivo*, a hydrogel delivering PDLSC-Exos significantly improved bone formation in rats with alveolar bone defects, in comparison with control and hydrogel-only treatments. Table 6 and Fig. 2 (top right panel) represent the role of PDLSC-Exos via delivery by biomaterial scaffolds.

**Table 6:** Summary of studies focused on the role of PDLSC-Exos in bone regeneration via delivery by biomaterial scaffolds

Study	Biomaterial scaffold	Mechanism	Outcome
Periodontitis			
Lei et al. [28]	Matrigel, β-TCP scaffolds	PDLSC-Exos delivered via scaffolds enhanced bone regeneration in rat periodontitis models	Demonstrated potential as a cell-free therapy to repair inflammatory bone loss
Zhao et al. [16]	Matrigel	PDLSC-Exos immobilized in Matrigel improved cell infiltration and faster bone repair in rat calvarial defect models	Hydrogel-delivered PDLSC-Exos significantly **↑**osteogenesis in alveolar bone defects
Han et al. [102]	3D bioprinted scaffolds	GelMA scaffolds with PDLSC-Exos enhanced MSC attachment and differentiation into ligament, bone, and cementum	Outperformed scaffolds with gingival fibroblast exosomes or no exosomes in tissue regeneration
Orthodontic tooth movement			
Liu et al. [37]	Simvastatin encapsulation	Simvastatin-loaded PDLSC-Exos increased drug solubility and suppressed relapse after OTM via enhanced osteogenesis	**↓**Bone-resorptive lacunae **↑**Increased osteogenic markers; PDLSC-Exos blocked relapse independently
Other bone defects			
Isik et al. [41]	UV-responsive GelMA hydrogel	PDLF-Exos in GelMA enhanced osteodifferentiation in MSCs (upregulation of OSP, ALP, RUNX2) without growth factors	**↑**Bone mineralization and regeneration in rat calvarial defect models
Diomede et al. [103]	Collagen membrane (Evo)	Evo combined with PDLSCs and PEI-Exos demonstrated high biocompatibility and osteogenic potential; PEI-Exos upregulated BMP2 and BMP4	**↑**Bone healing in rat calvarial defect models
Pizzicannella et al. [77]	3D collagen membrane (3D-COL)	3D-COL enriched with PDLSCs and PEI-Exos enhanced osteogenic expression and angiogenic proteins such as VEGF	**↑**Bone regeneration and vascularization in rat calvarial defect models
Zhao et al. [104]	Hydrogel	Hydrogel delivering PDLSC-Exos improved bone formation in rats with alveolar bone defects compared to control	Highlighted utility for bone regeneration via hydrogel-based delivery systems

### Clinical trials and patents on stem cells-derived exosomes in periodontitis

While *in-vitro* and *in-vivo* investigations have assessed the effects of PDLSC-Exos on periodontal regeneration in various animal models, the availability of clinical trials addressing the management of periodontitis remains limited. A preliminary Phase 1 clinical trial (Egypt; NCT04270006) performed by Beni-Seuf University sought to evaluate the efficacy of incorporating exosomes derived from adipose-derived stem cells into SRP as an adjunctive treatment to treat periodontitis. Participants aged between 18 and 50 years with Stage-III or Stage-IV (advanced periodontitis) were recruited, alongside a control group comprising individuals without periodontitis. Nonetheless, specific details regarding the trial’s progression have not been disseminated. To date, no additional clinical trials have been recorded.

Some patents related to exosomes derived from stem cells in the management of periodontitis offer substantial evidence for clinical applications. John and colleagues (US15884921) presented a formulation comprising stem cell exosomes, which might enhance PDL repair by promoting the synthesis of type I collagen in PDL fibroblasts. Jiang and colleagues (CN202110125452.2) validated the potential of exosomes derived from stem cells from human exfoliated deciduous teeth to stimulate the proliferation and differentiation of BMSCs, while also establishing their capacity to promote alveolar bone regeneration within a periodontitis model. Xu and coworkers (CN202210351949.0) developed and presented a mineralized collagen gel infused with gingival MSCs-Exos and outlined a method for its formulation. This synthetic gel can be administered via injection into the region of bone defects associated with periodontitis, thereby facilitating local bone regeneration or expansion. Tian and colleagues (CN202010607634.9) developed and revealed an injectable pharmaceutical formulation containing dental follicle stem cell-Exos, which has been shown to be effective in the treatment of periodontitis.

In a pioneering first-in-human case report published by our research group [109], horizontal ridge augmentation was successfully performed using a novel combination of bone allografts, platelet-rich fibrin (PRF), and a specialized subset of exosomes (Periosomes; exosomes derived from purified amniotic fluid). While not derived from PDLSCs, Periosomes represent a distinct class of regenerative exosomes. The case demonstrated progressive and substantial alveolar bone regeneration, indicating that Periosomes, when incorporated into enriched sticky bone with allografts and PRF, are a safe and highly promising biological agent for guided bone regeneration [109].

## Discussion

### Cell-free exosomal therapy versus cell-based therapies

One domain that has attracted considerable attention is the juxtaposition of exosome treatment versus cell-based interventions has been debated recently. Exosomes present numerous benefits in this context, with three pivotal benefits including (i) exosomes possess a small size that permits traversal across various minor barriers, such as the blood–brain barrier (BBB), which is not the case for cells; (ii) exosomes exhibit user-friendliness and enhanced storage feasibility; and (iii) exosomes do not provoke immune rejection since they do not possess MHC class I or II receptors, a primary drawback associated with stem cells [28].

Recently, debates surrounding stem cells have taken center stage in regenerative medicine, led by Dr. Arnold Caplan, the pioneer of MSCs [110]. For over three decades, MSCs have been recognized for their ability to differentiate into various tissue types [111]. However, Dr. Caplan later noted that this differentiation primarily occurred *in-vitro*, with limited clinical translation [112]. Subsequent discoveries revealed that the regenerative potential of MSCs lies predominantly in their secretory activity, which fosters an immunomodulatory environment conducive to tissue repair [113]. Further research identified exosomes secreted by MSCs as key players in establishing this microenvironment [114, 115]. Consequently, Dr. Caplan proposed renaming MSCs from “Mesenchymal Stem Cells” to “Medicinal Signaling Cells”, reflecting these pivotal insights [116].

Exosomes derived from stem cells, for example, hold the potential to facilitate angiogenesis and cell differentiation, as well as enhance cellular activity and survival. Furthermore, exosomes might expediently cultivate improved microenvironments by attenuating inflammatory responses. Cell-dependent interventions have faced criticism due to their protracted response times, which are particularly concerning for individuals requiring urgent medical intervention, such as those experiencing myocardial infarction. For example, upon intravenous injection of stem cells, several MSCs entering systemic circulation have been questioned with some studies’ findings that they may potentially result in entrapment within the pulmonary system [117]. Nevertheless, exosomes, owing to their small size, can circumvent the lungs and enter the bloodstream, thereby enabling more accurate targeting of tissues with an ability to target sites of inflammation [118]. Optimistically, the internal composition of exosomes might be substantially altered by factors such as mechanical stimulation, laboratory conditions, and cell sourcing, thereby presenting numerous opportunities [28].

Recent investigations have also indicated that exosome-based therapies hold greater promise as compared to stem cell-based treatments, attributed to their more precisely defined mechanisms [119]. The bilayer structure along with the presence of mRNAs, miRNAs, cytokines, chemokines, and immunomodulatory components, bestow PDLSC-Exos with exemplary tissue-targeting capabilities, biocompatibility, and pharmacokinetic characteristics [120–123]. Moreover, many studies have demonstrated that exosomes can mitigate inflammation, regulate cell proliferation, and expedite the healing of damaged tissues [122, 124]. Exosomes have been shown to improve viral infections [125), the immune system [126], lung [127], kidney [128], liver [129], heart [130], nerve [131], skin [132] as well as muscle and bone [133–135]. Furthermore, the capacity of exosomes to aid in both the detection and resolution of immune rejection and tumorigenicity linked to cell therapy, alongside the simplicity and non-invasive nature of exosome acquisition without the intricacies of cell isolation and potential damage, has broadened the scope and clinical application of exosome-based interventions [121, 136].

In summary, it has been previously documented that exosomes are favored over cell-based therapies for five distinct reasons: (i) these nanoscale vesicles are capable of being preserved for extended durations without diminishing their potential to enhance the immune response; (ii) exosomes exhibit superior compatibility with target cells compared to soluble factors produced by cells due to their surface characteristics, which closely resemble those of endogenous cells [137]; (iii) given their significantly smaller size relative to cells, exosomes can traverse capillaries and various biological barriers, including BBB, with greater ease [138]; (iv) exosomes lend themselves a straightforward manipulation and engineering processes [139]; and (v) the administration of exosomes is less complex than that of cell-based interventions, with the added possibility of nasal delivery [139].

### Factors affecting the integration of PDLSC-exos into routine clinical practice

#### Technical and biological variability in exosomes and MSC-based approaches

A critical limitation inherent to exosome-based research lies in the technical and biological variability associated with the extraction and characterization of both MSCs and their secreted EVs, especially exosomes [140]. The features of MSCs, and by extension, the cargo profile and bioactivity of their exosomes, are highly sensitive to the donor-specific and source tissue factors [141]. For instance, MSCs derived from PDL might vary considerably in their functional output and phenotype based on whether they are extracted from premolars versus molars, maxillary versus mandibular teeth, or from functionally active versus non-functional teeth [142, 143]. These physiological and anatomical variations may affect stem cell behavior through differences in local microenvironmental cues, vascularity, mechanical loading history, and developmental origin [144]. Exosomes sourced from the heterogeneous MSC populations are unlikely to be functionally or compositionally similar [145]. Variations in exosomal cargo may have significant downstream effects on target cell modulation, especially in assays assessing proliferation, osteogenesis, or immunomodulatory potential [146]. This variability introduces a major confounding factor that can obscure mechanistic interpretation, undermine reproducibility, and complicate inter-study comparisons. In clinical aspects, such heterogeneity also raises concerns associated with batch-to-batch consistency and therapeutic predictability [140].

#### Quantitative exosome characterization in functional studies

Another critical consideration in exosome-based research is the quantitative and biophysical characterization of the vesicles utilized in functional assays [147]. The mean diameter and particle number of exosomes are vital parameters that directly affect their biological activity, cellular uptake efficiency, and mechanistic interpretation [148]. Depending only on total protein concentration as a dosing metric may be misleading, as it does not account for differences in size distribution or particle concentration among varying preparations [149]. For example, two exosome specimens with similar protein cargo may vary significantly in the actual number of vesicles or their average diameter, potentially causing discrepancies in cellular responses [149]. Hence, it is recommended that incorporating absolute particle counts together with protein concentrations would provide a more precise and reproducible method for standardizing exosome dosage. Additionally, variations in mean diameter, even within the usual 30 to 150 nm range, may reflect shifts in vesicle subtype composition or altered cargo loading, both of which can affect downstream functional results [150]. Hence, we recognize the significance of incorporating both qualitative (marker expression, morphology, size) and quantitative (particle number) parameters into exosome characterization, and recommend their inclusion in future investigations to strengthen the rigor of experimental conclusions.

#### Standardization and quality control

Achieving consistent and reproducible isolation of high-quality PDLSC-Exos that are precisely specific/designed exosomal content has been the main challenge faced thus far. Various isolation techniques might produce an exosome population that may exhibit completely different characteristics. A thorough characterization of PDLSC-Exos, encompassing parameters such as size, miRNA profiles, protein composition, and morphology, is essential for maintaining quality control. The establishment of standardized protocols is imperative to guarantee reliable and consistent outcomes. The properties of PDLSC-Exos might fluctuate based on the donor’s age, health condition, and the culture environment of the donor cells. The formulation of standardized protocols for PDLSCs isolation and culture is vital for ensuring uniform exosome production.

#### Biological variability and individual responses

The biological influences and composition of PDLSC-Exos might differ markedly among individuals because of environmental and genetic influences. The therapeutic response to PDLSC-Exos treatment might also exhibit considerable variability across patients, influenced by factors such as individual immune responses, coexisting health conditions, and disease severity.

#### In-vivo delivery and targeting

Effectively administering PDLSC-Exos to targeted sites within the periodontal tissues presents significant challenges. Monitoring the retention and biodistribution of PDLSC-Exos *in-vivo* is essential for better understanding their therapeutic efficacy and potential adverse effects. Formulating strategies for the targeted delivery of PDLSC-Exos to specific cells or tissues within the periodontal microenvironment could enhance their therapeutic potential while reducing off-target effects.

#### Potential for adverse effects

Although generally regarded as immunologically inert, there exists a potential for immune responses to PDLSC-Exos, particularly in individuals with compromised immune systems. Despite the promising therapeutic benefits of PDLSC-Exos, the possibility of unintended effects on other organs or tissues cannot be entirely excluded. There is still a scarcity of data regarding the long-term efficacy and safety of PDLSC-Exos in clinical applications.

#### Regulatory and clinical translation

There exists considerable *in-vitro* and *in-vivo* evidence concerning the therapeutic potential of PDLSC-Exos for the treatment of a variety of human diseases [26]. Nonetheless, their efficacy is predicated more on their production processes than on their medical application. For the generation of clinically viable therapeutic PDLSC-Exos enhancements in chemistry, manufacturing, and controls (CMC) are imperative to adhere to Good Manufacturing Practices (GMP). This encompasses the establishment of a master cell bank, the formulation of methodologies for large-scale exosome synthesis and isolation, and the development of quality control and analytical methods for the manufacturing of therapeutic PDLSC-Exos. Given that the exosomes represent a relatively novel therapeutic modality, a universally recognized framework for their CMC has yet to be established. Importantly, the construction and execution of rigorous clinical trials aimed at assessing the efficacy and safety of PDLSC-Exos therapy in periodontal conditions is of utmost importance.

#### Manufacturing and scale-up

To manufacture exosomes on both large and small scale, various cellular lineages have been cultured utilizing dynamic or fixed culture systems including bioreactors and flasks [151–154]. Consequently, a rigorous and effective isolation and purification protocol is essential to achieve a high yield of uncontaminated PDLSC-Exos [155]. Despite multiple studies highlighting inconsistencies across different isolation methods and disparities in PDLSC-Exo recovery, there remains a pressing demand for technical advancements. Furthermore, the substantial costs associated with commercialization, coupled with the absence of insurance coverage and regulatory oversight from governmental entities, including FDA/CE approval for PDLSC-Exos, have significantly impeded the trajectory towards widespread commercialization.

#### Understanding mechanisms of action

The exact mechanisms through which PDLSC-Exos exert their therapeutic benefits are not fully elucidated. Further investigations are necessary to clarify the cellular and molecular mechanisms involved.

Hence, addressing these challenges will be pivotal for the effective translation of PDLSC-Exos therapy from experimental settings to clinical practice and its eventual implementation in the management of medical and dental conditions, including periodontitis.

### Limitations of included studies

#### Methodological heterogeneity

A significant limitation lies in the considerable methodological heterogeneity among the included *in-vitro* and *in-vivo* studies, which poses challenges in drawing definitive conclusions and identifying clear mechanistic pathways. While all studies focused on the application of PDLSC-Exos, several variables, especially those associated with exosome isolation approaches, culture protocols, and cell sources, introduce inconsistencies that may influence comparability and reproducibility. Among the *in-vitro* studies, the source of PDLSCs varied widely, such as premolars, third molars, and even donor-derived and commercially obtained cells, each potentially representing differences in donor profiles, microenvironment, and developmental stages. Moreover, culture conditions differed regarding basic media (i.e., α-MEM versus DMEM), FBS concentrations, and osteoinductive supplements (including ascorbic acid, β-glycerophosphate, and dexamethasone), all of which can change both exosomal cargo and cellular phenotype.

Exosome isolation and characterization approaches further contributed to the heterogeneity [140]. As discussed earlier, while differential ultracentrifugation was the most frequently used approach, other studies used size-exclusion chromatography, ultrafiltration, or polymer-based precipitation, each differing regarding the purity, yield, and the risk of co-isolating protein aggregates or non-exosomal vesicles. While numerous studies reported NTA data, not all provided particle concentration or size distribution, limiting the ability to correlate dose–response effects. Furthermore, the inconsistent utilization of normalization approaches (such as using particle count versus protein concentration) may affect biological results and limit reproducibility across studies. A diverse range of functional assays was utilized to assess exosome bioactivity, such as CFU, EdU, XTT, CCK-8, and MTT for proliferation; scratch wound assay for migration; ALP and Alizarin red staining for osteogenesis; and several protein/gene expression analyses using qRT-PCR, ELISA, and western blotting. While each of these approaches offers valuable insights, their varying sensitivities, endpoints, and interpretation criteria may complicate comparisons between studies assessing similar outcomes.

In the *in-vivo* studies, methodological variation was also evident in the choice of animal models (i.e., C57BL/6 J mice, Wistar versus Sprague–Dawley rats), their sex, weight, and age, together with the anatomical sites of induced defects (i.e., calvarial, periodontal). Experimental groups varied in their application of exosomes alone or in conjunction with bioactive molecules, scaffolds, or delivery systems, including liposomes or hydrogels. Evaluation methods ranged from μ-CT for structural assessment to immunohistochemistry and molecular assays for detecting inflammatory and osteogenic markers. The heterogeneity in sample sizes and outcome measures further limits the ability to synthesize quantitative conclusions across studies.

#### Variability in exosomal cargo

Another critical limitation to consider is the potential variability in exosomal cargo derived from the same cell type at varying passage numbers. While the included studies mainly utilized PDLSCs as the parent cell population, the passage range varied significantly, from early (P2-P3) to late passages (P8-P10). It is well established that MSCs undergo functional and phenotypic alterations with extended passages, such as senescence-related transitions in secretory profiles, changed differentiation ability, and decreased proliferation [156, 157]. These alterations inevitably affect the composition of secreted exosomes, such as variations in their cytokine, lipid, protein, and miRNA content [158]. Moreover, variations in external stimuli, including osteoinductive supplements in the culture medium, serum type, and oxygen tension, can modulate cellular signaling pathways and, in turn, change the molecular cargo loaded into exosomes [159]. Such variability can significantly influence the functionality of exosomes, whether pro-regenerative, immunomodulatory, or osteogenic [160], hence limiting the comparability and reproducibility of outcomes across studies. Despite the uniform cell source (PDLSCs), these dynamic changes in exosome content highlight the requirement for more rigorous control and reporting of culture parameters and passage numbers.

#### Exosome purity and requirements for clinical-grade production

Another important limitation relates to the purity of exosome preparations and the need for clinical translation. Several included studies used standard FBS as a supplement during cell culture, which is known to contain bovine-derived EVs [161, 162] that can co-isolate with target exosomes and contaminate the final product. Unless exosome-free FBS or fully exosome-free media is utilized, there remains a significant risk of introducing non-human vesicular components, potentially confounding experimental outcomes and compromising the specificity of observed biological effects [162]. This issue is especially relevant when attempting to quantify or characterize exosomal cargo, as lipid, RNA, or protein profiles from contaminating vesicles may be inadvertently attributed to the PDLSCs-Exos. Moreover, from a translational standpoint, the production of therapeutic-grade exosomes mandates compliance with GMP standards. Several current *in-vitro* and *in-vivo* studies were performed in research laboratories or academic settings without GMP oversight, raising concerns about the sterility, scalability, and reproducibility of exosome products intended for human application. Future research must prioritize the implementation of exosome-free culture systems and GMP-compliant workflows to ensure both scientific rigor and the feasibility of safe clinical application.

#### Variability in exosome concentration

A notable limitation in current exosome research is the extensive variability in the concentrations of exosomes used across *in-vitro* and *in-vivo* studies. This heterogeneity in dosing not only complicates direct comparisons between studies but also hinders the establishment of a clear dose–response relationship or therapeutic window. Moreover, the methods used to define and quantify exosome concentrations, whether by total protein amount, particle number, or volume, differ widely and are inconsistently reported. This lack of standardization raises concerns about reproducibility and makes it difficult to determine the optimal exosome dose for a specific biological outcome. For clinical translation, a more uniform and biologically relevant dosing strategy, potentially based on particle number normalized to cell count or surface markers, is essential to ensure consistency, safety, and efficacy of exosome-based therapies.

## Conclusion

In the present review article, PDLSC-Exos demonstrated significant therapeutic efficacy in both bone and periodontal regeneration as well as various medical conditions through the enhancement of cellular proliferation, osteoblast differentiation, and the modulation of inflammatory responses. These exosomes function by regulating miRNA and activating essential signaling pathways, thereby facilitating periodontal/bone regeneration, angiogenesis, and tissue repair in disorders such as periodontitis, OTM, and skeletal bony defects. Furthermore, they exhibited anti-inflammatory properties, leading to improved outcomes under inflammatory conditions such as periodontitis, IBD, and MS. Moreover, PDLSC-Exos played a role in anti-microbial and anti-cancer interventions, demonstrating their diverse applicability. The cell-free nature of these therapeutic agents makes PDLSC-Exos a versatile and promising tool for regenerative medicine and immune system regulation. The cell-free nature of these therapeutic agents positions PDLSC-Exos as a promising and adaptable instrument for regenerative medicine and immune system modulation.

Notwithstanding, the encouraging results from pre-clinical investigations have had slow progress towards the translation to clinical application, impeded by issues related to standardization, scalability of manufacturing processes, targeted delivery mechanisms, and compliance with regulatory frameworks. Existing patents emphasize innovative formulations and delivery methodologies, highlighting the necessity for comprehensive clinical trials to ascertain safety, efficacy, and long-term benefits. Tackling these obstacles is essential for the incorporation of PDLSC-Exos into the standard of clinical practice, which has the potential to revolutionize periodontal treatment and extend applications into the broader field of regenerative medicine.

## Supplementary Information

Below is the link to the electronic supplementary material.

Supplementary file1 (DOCX 15 KB)

Supplementary file2 (DOCX 40 KB)

Supplementary file3 (DOCX 27 KB)

## Data Availability

No datasets were generated or analysed during the current study.

## Abbreviations

*3D**-COL*: Three-dimensional collagen
*AFM*: Atomic force microscopy
*AKT*: AKT serine/threonine kinase
*ALP*: Alkaline phosphatase
*Amph**B*: Amphotericin B
*ANXA*: Annexin
*APAF-1*: Apoptotic Protease Activating Factor 1
*aPDT*: Antimicrobial photodynamic therapy
*ARG-1*: Arginase-1
*ARS*: Alizarin Red Staining
*ARSE*: Arylsulfatase E
*Asc.** Acid*: Ascorbic acid
*BAX*: BCL2-Associated X Protein
*BCL2*: B-cell lymphoma 2
*BMP*: Bone Morphogenetic Protein
*BMPR1A*: Bone Morphogenetic Protein Receptor Type 1A
*BMSCs*: Bone marrow mesenchymal stem cells
*CAP*: Cyclase-Associated Protein
*CCK-8*: Cell Counting Kit-8
*CCND1*: Cyclin D1
*CCR7*: C-C Chemokine Receptor Type 7
*CDH11*: Cadherin 11
*CEMP1*: Cementum Protein 1
*c-Fos*: Cellular proto-oncogene Fos
*CGC*: Conjunctival goblet cells
*COL1A1*: Collagen type I Alpha 1 Chain
*Cryo-EM*: Cryo-Electron Microscopy
*CS*: Citrate synthase
*CS*: Citrate Synthase
*Cur*: Curcumin
*Dc-Stamp*: Dendrocyte-expressed seven transmembrane protein
*Dex*: Dexamethasone
*DLR Assay*: Dual-Luciferase Reporter Assay
*DLS*: Dynamic Light Scattering
*DMEM*: Dulbecco’s modified eagle medium
*DSS*: Dextran sodium sulfate
*EGFR*: Epidermal Growth Factor Receptor
*ELISA*: Enzyme-linked immunosorbent assay
*Emo@PDL-Exos*: Periodontal ligament stem cells-derived exosome-loaded emodin
*eNOS*: Endothelial nitric oxide synthase
*ERK*: Extracellular signal-regulated kinase
*EVs*: Extracellular vesicles
*EVs-ASP*: Aspirin-loaded vesicles
*FABP4*: Fatty Acid Binding Protein 4
*FBS*: Fetal bovine serum
*FCM*: Flow Cytometry
*FGF*: Fibroblast Growth Factor
*FOXO1*: Forkhead Box O1
*FOXP3*: Forkhead Box P3
*FTIR*: Fourier Transform Infrared Spectroscopy
*GA*: Gallic acid
*GelMA*: Gelatine methacrylate
*GSK-3β*: Glycogen synthase kinase-3 beta
*GTFB*: General Transcription Factor B
*H&E*: Hematoxylin and eosin
*hAMSCs*: Human adipose mesenchymal stem cells
*hBFP-MSCs*: Human buccal fat pad-derived mesenchymal stem cells
*HEPES*: 4-(2-Hydroxyethyl)-1-piperazineethane sulfonic acid
*HO-1*: Heme Oxygenase 1
*hPDL*: Human periodontal ligament
*HPLC*: High-Performance Liquid Chromatography
*CFU*: Colony forming unit
*HSP90*: Heat Shock Protein 90
*HUVECs*: Human umbilical vein endothelial cells
*IBSP*: Integrin Binding Sialoprotein
*IDH*: Isocitrate Dehydrogenase
*IGF*: Insulin-like Growth Factor
*IHC*: Immunohistochemistry
*IL*: Interleukin
*Inos*: Inducible Nitric Oxide Synthase
*i-PDLSCs*: Inflammatory PDLSCs
*KEAP1*: Kelch-like ECH-associated protein 1
*L. acidophilus*: *Lactobacillus acidophilus*
*LC**–**MS/MS*: Liquid Chromatography-Tandem Mass Spectrometry
*LDH1*: Lactate dehydrogenase 1
*L-Glut*: L-Glutamine
*LPS*: Lipopolysaccharide
*MB staining*: Methylene blue staining
*MGP*: Matrix Gla Protein
*MINCLE*: Macrophage Inducible C-Type Lectin
*MINPP*: Multiple Inositol-Polyphosphate Phosphatase 1
*MiRNA*: Micro-RNA
*MMP*: Matrix Metallopeptidase
*MRC1*: Mannose Receptor C-Type 1
*MSCs*: Mesenchymal stem cells
*MSCGM-CD*: Mesenchymal stem cell growth medium chemically defined
*MSX1*: Msh Homeobox 1
*MTT Assay*: Methylthiazolyldiphenyl-tetrazolium Bromide Assay
*IFS*: Immunofluorescence;
*MUC5AC*: Mucin 5AC
*NA*: Not associated
*NALP3*: Nucleotide-binding oligomerization domain, leucine-rich repeat and pyrin domain-containing 3
*NDUFB3*: NADH:Ubiquinone Oxidoreductase Subunit B3;
*NEAA*: Non-essential amino acids
*NFATc1*: Nuclear factor of activated T-cells, cytoplasmic 1
*NF-κB*: Nuclear Factor Kappa-Light-Chain-Enhancer of Activated B Cells
*NGS*: Next generation sequencing
*NQO-1*: NAD(P)H Quinone Dehydrogenase 1
*NR*: Not reported
*NRF2*: Nuclear Factor Erythroid 2-Related Factor 2
*NTA*: Nanoparticle Tracking Analysis
*OCN*: Osteocalcin
*OD*: Osteogenic differentiation
*OGDH1*: Oxoglutarate dehydrogenase 1
*OPN*: Osteopontin
*OSX*: Osterix
*OTM*: Orthodontic tooth movement
*P2RX7*: Purinergic Receptor P2X7
*PCNA*: Proliferating Cell Nuclear Antigen
*PDGF*: Platelet-Derived Growth Factor
*PDLFs-Exos*: Exosomes derived from periodontal ligament fibroblasts
*PDLSCs-CM*: Conditioned medium from PDLSCs
*PDLSC-Exos*: Periodontal ligament stem cell exosomes
*PEI*: Polyethylenimine-engineered extracellular vesicles
*Pen*: Penicillin
*PRF*: Platelet-rich fibrin
*PGE2*: Prostaglandin E2
*PPARγ*: Peroxisome Proliferator-Activated Receptor Gamma
*qRT-PCR*: Quantitative Reverse Transcription Polymerase Chain Reaction
*RAB27*: Ras-related protein Rab-27;
*RANKL*: Receptor Activator of Nuclear Factor Kappa-B Ligand
*RORC*: RAR-related Orphan Receptor C
*RORγτ*: RAR-related Orphan Receptor Gamma T
*RR-MS*: Relapsing-Remitting multiple sclerosis
*RUNX2*: Runt-related transcription factor 2
*S. mutans*: *Streptococcus mutans*
*SATB2*: Special AT-rich sequence-binding protein 2
*SD rats*: Sprague-Dawley rats
*SDHB*: Succinate Dehydrogenase Complex Iron-Sulfur Subunit B
*SEM*: Scanning Electron Microscopy
*SEVs*: Smal extracellular vesicles
*SIPA*: Signal-Induced Proliferation-Associated
*SM-Exos*: Exosomes from strained microenvironment
*Smurf1*: SMAD ubiquitination regulatory factor 1
*SOX9*: SRY-Box Transcription Factor 9
*SPARC*: Secreted Protein Acidic and Rich in Cysteine
*SRP*: Scaling and root planning
*Strep*: Streptomycin
*TEM*: Transmission Electron Microscopy
*TGFBR*: Transforming Growth Factor Beta Receptor
*TGF-β1*: Transforming growth factor beta 1
*Th17*: T helper 17
*TMCO1*: Transmembrane and Coiled-Coil Domain Containing 1
*TNF-α*: Tumor necrosis factor alpha
*TRAF6*: TNF receptor-associated factor 6
*TRAP*: Tartrate-resistant acid phosphatase
*Treg*: Regulatory T cells
*TUFT1*: Tuftelin 1
*TWIST1*: Twist Family BHLH Transcription Factor 1
*VDR*: Vitamin D Receptor
*VEGFB*: Vascular Endothelial Growth Factor B
*WB*: Western Blotting
*WNT*: Wingless-type
*XBP1*: X-box Binding Protein 1
*α-MEM*: Minimum essential medium eagle-alpha modification
*Βgp*: Beta-glycerophosphate
*μ-CT*: Micro-Computed tomography
