# Supplementary material for: Mechanistic insights into periodontal ligament stem cell-derived exosomes in tissue regeneration
Source: Clin Oral Investig. 2025 Jun 25;29(7):357. doi: 10.1007/s00784-025-06422-1 (PMC12198077; doi:10.1007/s00784-025-06422-1)
Supplement: Supplementary file 1 — Supplementary file1 (DOCX 15 KB) [file 784_2025_6422_MOESM1_ESM.docx]

**Table S1**. Literature search strategy.

| **Search term (Title, Abstract, and Keywords)** | **Scopus** | **Web of Science** | **PubMed** | **Total** |
| --- | --- | --- | --- | --- |
| “Exosome*” AND “Periodontal Ligament” AND “Stem Cell*” | 163 | 81 | 66 | 310 |
| “Exosomal” AND “Periodontal Ligament” AND “Stem Cell*” | 24 | 18 | 20 | 62 |
| “Extracellular Vesicle*” AND “Periodontal Ligament” AND “Stem Cell*” | 76 | 51 | 48 | 175 |
| “EVs*” AND “Periodontal Ligament” AND “Stem Cell*” | 39 | 29 | 23 | 91 |
| **Total** | 302 | 179 | 157 | 638 |
