# Supplementary material for: Mechanistic insights into periodontal ligament stem cell-derived exosomes in tissue regeneration
Source: Clin Oral Investig. 2025 Jun 25;29(7):357. doi: 10.1007/s00784-025-06422-1 (PMC12198077; doi:10.1007/s00784-025-06422-1)
Supplement: Supplementary file 2 — Supplementary file2 (DOCX 40 KB) [file 784_2025_6422_MOESM2_ESM.docx]

**Table S2**. Primary characteristics of the included *in-vitro* studies.

| **Study & Country** | **Field (Disease)** | **Cell Source; Passage** | **Cell Culture & Osteogenic Differentiation Media Composition** | **Exosome isolation method** | **Exosome concentration** | **Target Osteogenic Markers** | **Assays** | **Main Findings** |
| --- | --- | --- | --- | --- | --- | --- | --- | --- |
| Lei et al. 2022 (32); China | Periodontitis | Premolars & 3^rd^ Molars; P3 | Cell culture: DMEM, 20% FBS, 100 U/mL Pen, 100 µg/ml Strep  OD: DMEM, 10% FBS, 10 mM βGP, 50 μM Asc. acid, 0.1 μM Dex | Polymer-based precipitation  Differential ultracentrifugation | 1, 5, 10 μg/mL | RUNX2, OCN, OSX, WNT1, WNT3a, WNT10a, β-catenin, GSK-3β | CCK8 assay, Cell scratch assay, ARS, TEM, NTA, FCM, WB, PKH26 staining, qRT-PCR | PDLSC-Exos led to an increase in the formation of mineralized nodules and the expressions of osteogenic genes and proteins in i-PDLSCs; PDLSC-Exos suppressed the over-activation of canonical Wnt signaling to recover the OD capacity of i-PDLSCs. |
| Shi et al. 2023 (33); China | Periodontitis | Donated; NR | Cell culture: α**-**MEM, 10% FBS, 0.292 mg/mL L-Glut, 100 U/mL Pen, 100 mg/mL Strep  OD: NR | Differential ultracentrifugation | 2 μg/mL | TNF-α, ARG-1, IL-10, IL-6, IL-1β, LDH1, CS, OGDH1 | ARS, DLS, SEM, WB, Seahorse metabolic flux assay, HPLC, Lactate and ATP assay | EVs-ASP inhibited the inflammatory environment of LPS-induced macrophage, and promoted anti-inflammatory macrophages formation |
| Lin et al. 2022 (29); China | Periodontitis | Premolars; P3 | Cell culture: α**-**MEM, 15% FBS, 1% Pen/Strep  OD: 10% FBS, 10 mmol βGP, 10 nmol Dex, 50 μg/ml Asc. acid | Differential ultracentrifugation | 0.05 μg/mL | IL-6, IL-8, RUNX2, OPN, miR-34c-5p, Calnexin, SATB2, ERK1/2, p-ERK1/2 | ALP/ARS staining, CFU assay, FCM, TEM, NTA, WB, DLR assay, CCK8 assay, qRT-PCR | PDLFs-Exos induced by PGE2 contained up-regulated miR-34c-5p in comparison with non-stimulate PDLFs. Meanwhile, miR-34c-5p inhibited OD of PDLSCs via SATB2/ ERK. |
| Wang et al. 2023 (34); China | Periodontitis | Premolars & 3^rd^ Molars; P3 | Cell culture: DMEM, 20% FBS, 100 U/mL Pen, 100 µg/ml Strep  OD: DMEM, 10% FBS, 10 mM βGP, 50 μM Asc. acid, 0.1 μM Dex | Polymer-based precipitation  Differential ultracentrifugation | 150 µg/mL | RUNX2, OCN, COL1A1, ALP, SMAD1, SMURF1 | PKH26 staining, qRT-PCR, WB, NTA | PDLSCs stimulated by strain can secrete functional Exos that can be taken up by BMSCs and promote OD of BMSCs. |
| Isik et al. 2023 (35); Turkey | Bone defects | Donated; NR | Cell culture: DMEM, 10% FBS, 1% Pen/Strep, 1% NEAA  OD: NR | Differential ultracentrifugation | 10, 100 µg/mL | RUNX2, ALP, OSP | DLS, TEM, qRT-PCR | hAMSC) on GelMA/hPDLFs-Exs hydrogels begin to up-regulate RUNX2, ALP, and OSP expressions, that support the hypothesis of growth factors-free induction of OD in hMSCs. |
| Liu et al. 2022 (36); Iraq | Orthodontic tooth movement | Premolars & 3^rd^ Molars; P3-P5 | Cell culture: α**-**MEM, 15% FBS  OD: DMEM, 10% FBS, 10 mM βGP, 50 μM Asc. acid, 0.1 μM Dex | Differential ultracentrifugation | NR | RUNX2, ALP, β-catenin, OSX, COL1A1 | ARS, FCM, TEM, WB, qRT-PCR | The expression level of osteogenic-related genes and proteins in the exosomal simvastatin group is higher than in other groups. |
| Tang et al. 2023 (37); China | Inflammatory bowel disease | NR; NR | Cell culture: α-MEM, 10% FBS 1% Pen/Strep  OD: NR | Differential ultracentrifugation | 200 µg/mL | IL-6, IL-10, IL-23, IL-17A, TGF-β1 | EdU cell proliferation assay, MTT assay, NTA, FCM, WB, IFS analysis | EVs derived from thermally activated MSCs exhibited high expression of PD-L1, which modulated Th17/Treg cell differentiation through the PD-1/PD-L1 signaling pathway and played a significant anti-inflammatory role in DSS-induced colitis. |
| Xie et al. 2024 (38); China | Periodontitis | 3^rd^ Molars; P3 | Cell culture: α-MEM; 20% FBS, 100U/mL Pen, 100 mg/mL Strep, 0.25 mg/mL Amph B  OD: DMEM, 15% FBS 100 nM Dex, 10 mM βGP, 200 mM Asc. acid | Differential ultracentrifugation | 500 µg/mL | TRAP, TRAF6, NFATc1, c-Fos, NF-κB, AKT, GSK-3β | FCM, TEM, NTA, WB, ALP staining, ARS, qRT-PCR, PKH67 staining | Exosomal circ_0000722 from PDLSCs undergoing OD might promote osteoclastogenesis by upregulating TRAF6 expression and activating downstream NF-κB and AKT signaling pathways. |
| Huang et al. 2022 (39); China | Orthodontic tooth movement | Premolars | Cell culture: α-MEM; 15% FBS  OD: NR | Differential ultracentrifugation | 50 µg/mL | Annexin III, Annexin VI, ERK, RAB27A/B, Dc-Stamp, MMP9 | PKH26 staining, TEM, NTA, IFS analysis, WB, LC-MS/MS, qRT-PCR | Mechanical force treatment promotes the biogenesis of PDLSC-Exos and increases ANXA3 to facilitate exosome internalization, which activates ERK phosphorylation, thus inducing osteoclast differentiation. |
| Diomede et al. 2018 (30); Italy | Bone defects | Premolars & 3^rd^ Molars; P2 | Cell culture: DMEM, 10% FBS, 1% Pen/Strep, 1% fungizone  OD: NR | Polymer-based precipitation | 5000, 500, 100, 50, 25 μg/mL | ALPL, ARSE, BMP1, BMP6, BMPR1A, CDH11, COL11A1, COL12A1, COL14A1, COL15A1, COL16A1, COL1A1, COL1A2, COL4A3, COL4A4, EGFR, FGF1, FGF2, IBSP, IGF1, IGF1R, IGF2, MGP, MINPP, MMP2, MMP8, MSX1, PDGFA, PDGF4, RUNX2, SMAD1-7, SMAD9, SOX9, SPARC, TGFB1, TGFB2, TGFB3, TGFBR1, TGFBR2, TUFT1, TWIST1, VDR, VEGFB | FCM, DLS, AFM, WB, ARS, PKH26 staining, qRT-PCR | Evo enriched with EVs and PEI-EVs showed high biocompatibility and osteogenic properties; The study demonstrated the upregulation of osteogenic genes, such as TGFB1, MMP8, TUFT1, TFIP11, BMP2, and BMP4, in the presence of PEI-EVs. |
| Zheng et al. 2023 (40); China | Orthodontic tooth movement | Premolars & 3^rd^ Molars; P3-P4 | Cell culture: DMEM; 10% FCS; 100 U/mL Pen/Strep  OD: NR | Polymer-based precipitation | NR | hsa-miR-126-5p, hsa-miR-1306-5p, hsa-miR-146a-5p, hsa-miR-99a-5p, hsa-miR-125a-5p, hsa-miR-140-3p, hsa-miR-21-5p, hsa-miR-23a-3p, hsa-miR-29a-3p, hsa-miR-485-3p | IFS analysis, NTA, TEM, WB, qRT-PCR, CCK8 assay | The expression levels of miRNAs secreted by PDLSC-Exos due to mechanical force were very different compared to PDLSC-Exos under non-mechanical stress. |
| Lan et al. 2023 (41); China | Periodontitis | Premolars & 3^rd^ Molars; P3 | Cell culture: DMEM, 10% FBS, 1% Pen/Strep  OD: DMEM, 10% FBS, 50 μM ascorbate-2-phosphate, 10 mM βGP, 100 nM Dex | Differential ultracentrifugation | 50 µg/mL | COL1, ALP, RUNX2, CCND1 | TEM, WB, CCK8 assay, ARS/ALP staining, qRT-PCR | Cur-PDLSC-EV can promote OD by activating Wnt/β-catenin, providing reference bases for the treatment of periodontal diseases. |
| Chiricosta et al. 2020 (42); Italy | Oncogenesis | Premolars & 3^rd^ Molars; P2 | Cell culture: MSCGM-CD Bulletin medium  OD: NR | Polymer-based precipitation | NR | miR-24, miR-142, miR-335, miR-296, miR-490 | NGS | PDLSC-Exos contain MIR24-2, MIR142, MIR296, MIR335, and MIR490, miRNAs regulating genes that are involved in “Ras protein signal transduction” and “Actin/microtubule cytoskeleton organization” processes that regulate cell growth and differentiation during cytokinesis. |
| Niu et al. 2024 (43); China | Periodontitis | Premolars; P2-P6 | Cell culture: DMEM; 10% FBS; 100 U/mL Pen/Strep  OD: DMEM, 10% FBS, 100 U/mL Pen/Strep, 10 nM Dex, 10 mM βGP 0.2 mM Asc. acid | Differential ultracentrifugation | 50 µg/mL | FOXO1, iNOS, Arg-I, IL-10, TGF-β, TNF-α, IL-1β, ALP, RUNX2, COL1 | FCM, TEM, WB, IFS staining, PKH26 staining, ARS, qRT-PCR | FoxO1-overexpressed Exos promoted OD of hPDLSCs in the inflammatory environment and polarized THP-1 cells from the M1 phenotype to the M2 phenotype. |
| Pizzicannella et al. 2019 (44); Italy | Bone defects | Premolars & 3^rd^ Molars; P2 | Cell culture: MSCGM-CD Bulletin medium  OD: NR | Polymer-based precipitation | 50 μg/mL | RUNX2, COL1A1, BMP2/4, VEGFA, VEGFR2 | FCM, DLS, AFM, WB, IFS staining, qRT-PCR, MTT assay | The study showed an increased expression of osteogenic markers in PDLSCs cultured with the 3D-COL and PEI-EVs, associated also with the increased VEGF and VEGFR2 levels. |
| Kang et al. 2018 (45); Korea | Periodontitis | Company; NR | Cell culture: α-MEM; 10% FBS; 1% Pen/Strep  OD: α-MEM, 10% FBS, 200 mM L-glut, 1% Pen/Strep, 10 nM Dex, 50 µg/mL Asc. acid, 5 mM βGP, 1.8 mM monopotassium phosphate | Ultrafiltration & Differential ultracentrifugation | NR | IL-6, TNF-α, IL-10 | FCM, NTA, ARS, qRT-PCR, ELISA | LPS-stimulated PDLSCs induce M1 polarization of macrophages via Exos, suggesting that PDLSC-Exos might be a potential therapeutic target for inflammation in the periodontium. |
| Wang et al. 2023 (46); China | Bone defects | Premolars & 3^rd^ Molars; P3-P5 | Cell culture: α-MEM  OD: α-MEM, 10% FBS, 50 mg/L Asc. acid, 10 mM βGP, 10 nM Dex | Ultrafiltration | 50 µg/mL | OPG, OCN, RUNX2, SP7, RANKL | FCM, TEM, WB, ARS, qRT-PCR | Cell culture density not only affects the osteogenic induction of stem cells themselves but may also affect the differentiation ability of BMSCs by secreting exosomes. |
| Zhang et al. 2020 (47); China | Periodontitis | Premolars; P2-P5 | Cell culture: α-MEM, 10% FBS, 100 μg/ml Pen, 100 U/ml Strep  OD: NR | Differential ultracentrifugation | 50 µg/mL | VEGFA | IFS staining, ARS, FCM, TEM, WB, DLR assay | Exosomes secretion of PDSLCs was augmented by inflammation, and promoted angiogenesis of HUVECs, whereas blocking secretion of exosomes led to degenerated angiogenesis of HUVECs. |
| Zhao et al. 2022 (19); China | Periodontitis | Premolars; P5 | Cell culture: α-MEM, 15% FBS, 0.1 mM Asc. acid, 1% L-glut, 1% antibiotic–antimycotic  OD: α-MEM, 15% FBS, 50 μg/mL Asc. acid, 100 nM Dex, 10 mM βGP | Differential ultracentrifugation | 1, 5, 10 μg/mL | FGF, PCNA, BCL2, ERK1/2, p-ERK1/2 | TEM, NTA, WB, CCK8 assay, Cell scratch assay, qRT-PCR | P-EVs accelerated the repair of bone defects, partially through promoting cell proliferation and migration. |
| Lu et al. 2023 (48); China | Periodontitis | Premolars; P2-P4 | Cell culture: DMEM, 10% FBS  OD: Stem Cell Osteogenic Induction Kit | Differential ultracentrifugation | 15 µg/mL | TRAF6, NFATC1, C-FOS, mi-R31-5p | ARS, IFS staining, FCM, TEM, WB, qRT-PCR, DLR assay | Exosomal miR-31-5p derived from PDLSCs regulates alveolar bone regeneration by targeting eNOS. |
| Chang et al. 2023 (49); China | Orthodontic tooth movement | Premolars; P5-P10 | Cell culture: α-MEM, 20% FBS, 1% Pen/Strep  OD: NR | Differential ultracentrifugation | 100 µg/mL | Untargeted mi-RNA | TEM, NTA, WB, PKH26 staining, MTS assay | The exosomes derived from cyclic tension-stretched PDLCs can promote the migration of BMSCs. |
| Kang et al. 2023 (50); China | Periodontitis | Company; P3-P6 | Cell culture: DMEM 10% FBS, 1% Pen/Strep  OD: NR | Differential ultracentrifugation | 10 µg/mL | XBP1, TNF-α, IL-1β, IL-6, RORγτ, IL-17A, FOXP3, IL-10 | NTA, TEM, WB, FCM, qRT-PCR, ELISA, DLR assay | Exo‐miR‐205‐5p derived from PDLSCs relieves the inflammation and balances the Th17/Treg cells in CP through targeting XBP1. |
| Han et al. 2023 (51); Australia | Bone defects | 3^rd^ Molars; P5 | Cell culture: DMEM, 10% FBS, Antibiotic–Antimycotic  OD: DMEM, FBS, 100 nM dex, 10 mM βGP, 50 μM Asc. acid | Size-exclusion chromatography | NR | RUNX2, BSP, COL1A1, PPARγ, FABP4 | NTA, TEM, ELISA, FTIR, FCM, Cell scratch assay, qRT-PCR | Exos from the three periodontal cells promoted proliferation, migration and osteogenic gene expression. |
| Zheng et al. 2019 (52); China | Periodontitis | Premolars; P3-P6 | Cell culture: DMEM, 10% FBS, 100 U/ml Pen, 100 μg/ml Strep  OD: NR | Differential ultracentrifugation | NR | FOXP3, Sirtuin-1, RORC, IL-10, IL-17, TNF-α, | FCM, IFS staining, WB, qRT-PCR, ELISA, CCK-8 assay, DLR assay, PKH67 staining | PDLSC-Exos alleviated inflammatory microenvironment through Th17/Treg/miR‐155‐5p/SIRT1 regulatory network. |
| Wu et al. 2023 (53); China | Periodontitis | Premolars; P4-P6 | Cell culture: DMEM, 10% FBS, 100 U/ml Pen, 100 μg/ml Strep  OD: NR | Ultrafiltration & polymer-based precipitation | 25, 50, 100 µg/mL | IL-1β, TNF-α, iNOS, IL-6, IL-10, Arg-1, | NTA, TEM, WB, FCM, ELISA, qRT-PCR, DLR assay | Stretching force stimulated PDLSCs to secrete exosomes and that exosomal miR-9-5p induced M1 polarization in macrophages and the release of IL-1b, TNF-a, and IL-6, through the SIRT1/NF-kB signaling pathway |
| Dai et al. 2022 (54); China | Periodontitis | Premolars & 3^rd^ Molars; P2 | Cell culture: DMEM 10% FBS, 1% Pen/Strep  OD: NR | Differential ultracentrifugation | 100, 200, 300 µg/mL | NDUFB-3, SDHB, CS, IDH | MTT assay, ELISA, qRT-PCR, ALP staining, ARS, WB, NTA | GA could improve the proliferative activity and oxidative stress level of i-PDLSCs, increase aerobic metabolism in cells, and improve the function of its exosome to contribute to OD. |
| Novello et al. 2022 (55); France | Periodontitis | 3^rd^ Molars; P3-P7 | Cell culture: DMEM, 10% FBS, 2 mmol/L L-glut, 20 mmol/L HEPES, 100 U/mL Pen, 100 µg/mL Strep  OD: DMEM, 10% FBS, 50 µg/mL Asc. acid, 10 mM βGP, 100 nM Dex | Polymer-based precipitation | 62.5, 125, 250, 500 µg/mL | HSP90, IL-6, IL-8, BSP | FCM, TEM, IFS staining, MTT assay, ELISA, qRT-PCR, ARS | This study showed an enhancement of Saos-2 proliferation under the influence of PDLSC-CM and an increase in some markers of osteoblastic differentiation, but this differentiation was not complete. |
| Zhao et al. 2022 (56); China | Bone defects | Premolars & 3^rd^ Molars; NR | Cell culture: α-MEM, 15% FBS, L-glut, 1% Pen/Strep  OD: α-MEM, 10% FBS, 10 mM βGP, 50 mg/L Asc. acid, 10 nM Dex, 1% Pen/Strep, 2 mM L-glut | Differential ultracentrifugation | 10, 20 µg/mL | NA | FCM, TEM, WB, NTA, CCK-8 assay, ARS, ALP staining | PDLSC-Exos significantly promoted the proliferation and OD of BMSCs. |
| Cui et al. 2023 (57); China | Periodontitis | Premolars & 3^rd^ Molars; P2-P4 | Cell culture: α-MEM  OD: NR | Differential ultracentrifugation | 10 µg/mL | IL-6, TNF-α, TGF-β, | TEM, WB, NTA, PKH67 staining, qRT-PCR | sEVs derived from LPS-preconditioning PDLSCs could significantly increase the expression of M1 markers and inflammatory cytokines, whereas decreased the expression of M2 markers and anti-inflammatory cytokines |
| Pourhajibagher et al. 2024 (58); Iran | Dental caries | 3^rd^ Molars; P2-P3 | Cell culture: DMEM, 10% FBS, 1% Pen/Strep  OD: NR | Polymer-based precipitation | 2, 4, 8, 16, 31, 63, 125, 250, 500, 1000 µg/mL | GTFB, SIPA | FCM, TEM, DLS, CFU assay, XTT assay, qRT-PCR | aPDT using Emo@PDL-Exo can effectively reduce the cell viability, biofilm activity, and metabolic potency of *S. mutans* and *L. acidophilus*. |
| Lu et al. 2023 (59); China | Periodontitis | Premolars; P4-P5 | Cell culture: α-MEM, 10% FBS, 1% antibiotic–antimycotic, 1% L-glut  OD: α-MEM, 10% FBS, 50 μg/mL Asc. acid, 100 nM Dex, 10 mM βGP | Differential ultracentrifugation | 1, 5, 10 µg/mL | RAB27A, COL1A1, ALP, RUNX2, BSP | FCM, TEM, SEM, qRT-PCR, WB, ARS, PKH26 staining | PDLSC-Exos enhanced OD of BMMSCs. |
| Soundra Rajan et al. 2017 (60); Italy | Multiple sclerosis | Premolars; P2 | Cell culture: MSCGM-CD Bulletin medium  OD: MSCGM-CD Bulletin medium | Differential ultracentrifugation | NR | NALP3, CASPASE 1, IL-1β | FCM, TEM, ELISA, WB | The secretory molecules present in CM and purified EMVs obtained from PDLSCs of RR-MS patients modulate NF-κB level and inhibit NALP3 inflammasome activation |
| Rajan et al. 2016 (61); Italy | Multiple sclerosis | Premolars; P2 | Cell culture: MSCGM-CD Bulletin medium  OD: MSCGM-CD Bulletin medium | Polymer-based precipitation | NR | RUNX2, ALP, FABP4, PPARγ | FCM, TEM, MTT assay, qRT-PCR, WB | hPDLSCs-CM and hPDLSCs-EMVs reduce pro-inflammatory cytokines IL-17, IFN-γ, IL-1β, IL-6, TNF-α, and induce anti-inflammatory IL-10. |
| Liu et al. 2020 (62); China | Bone defects | Premolars; P3-P5 | Cell culture: α-MEM, 10% FBS, 1% Pen/Strep  OD: α-MEM, 10% FBS, 10 mM βGP, 50 μM Asc. acid, 100 nM Dex | Differential ultracentrifugation | 50 µg/mL | ALP, RUNX2, SP7 | FCM, TEM, WB, NTA, ALP staining, ARS, qRT-PCR, PKH26 staining | Exosomal miRNAs derived from PDLSCs may promote OD of BMSCs |
| Liu et al. 2023 (63); China | Diabetes mellitus | 3^rd^ Molars; P2 | Cell culture: α-MEM, 10% FBS, 1% Pen/Strep  OD: α-MEM, 10% FBS, 10 mM βGP, 50 μM Asc. acid, 100 nM Dex | Differential ultracentrifugation | 20, 50, 100 µg/mL | NRF2, KEAP1, HO-1, NQO-1, HISTONE H3 | ARS, ALP staining, CFU assay, qRT-PCR, CCK-8 assay  Cell scratch assay, TEM, NTA, IFS staining, WB, DLR assay | High levels of glucose accelerate premature senescence in PDLSCs and that PDLSC-Exos promote the regeneration of senescent PDLSCs. |
| Han et al. 2024 (31); Australia | Periodontitis | 3^rd^ Molars; P5 | Cell culture: DMEM, 10% FBS, 1% Antibiotic-Antimycotic  OD: DMEM, 10 % FBS, 50 μg/ mL Asc. acid, 3 mM βGP, 10 nM Dex | Differential ultracentrifugation & SEC | 10^10^ particles/mL | RUNX2, BSP, ALP, CEMP1 | TEM, cryo-EM, FTIR, NTA, ELISA, FCM, ALP staining, ARS, qRT-PCR | 3D bioprinted GelMA/hPDLCs-sEVs scaffolds promote cell attachment, as well as ligamentous, osteogenic and cementogenic differentiation, of hBFP-MSCs |
| Ren et al. 2024 (64); China | Dry eye disease | Premolars & 3^rd^ Molars; P3 | Cell culture: DMEM, 10% FBS, 2 mmol/L L-glut, 100 U/ml Pen, 100 μg/ml Strep  OD: NR | Differential ultracentrifugation | NR | TNF-α, IL-10, ARG1, MUC5AC | FCM, TEM, WB, NTA, qRT-PCR, PKH26 staining | PDLSC-Exos could protect CGCs against M1 macrophage-mediated inflammation, and the protective effects of PDLSC-Exos are partly attributable to their effects on M1 macrophages. |
| Xu et al. 2022 (65); China | Orthodontic tooth movement | Premolars; P3-P5 | Cell culture: α-MEM, 20% FBS 1% Pen/Strep  OD: α-MEM, 10% FBS, 10 mM βGP, 50 μM Asc. acid, 100 nM Dex | Differential ultracentrifugation | 1, 5, 10 µg/mL | ALP, RUNX2, OCN | FCM, TEM, NTA, WB, CCK-8 assay, ALP staining, ARS, qRT-PCR | PDLSC-Exos effectively enhanced OTM and promoted osteogenesis on the tension side, including increasing trabecular bone parameters and promoting the expression of osteogenic-related biomarkers |
| Zhong et al. 2022 (66); China | Diabetes Mellitus | Stem cell bank; NR | Cell culture: α-MEM, 10% FBS, 1% Pen/Strep  OD: α-MEM, 10% FBS, 2 mM βGP, 100 μM Asc. acid, 10 nM Dex | Differential ultracentrifugation | NR | ALP, RUNX2, COL1A1, TMCO1, P2RX7, IL-1β, IL-6, IL-10, TNF-α | ALP staining, ARS, TEM, NTA, WB, IFS staining, qRT-PCR | Metformin could upregulate P2X7R-mediated exosome release and decrease intracellular miR-129-3p accumulation, which restores ER homeostasis and thereby rescues the impaired PDLSCs. |
| Yu et al. 2021 (67); China | Periodontitis | 3^rd^ Molars; P2-P5 | Cell culture: DMEM, 10% FBS, 1% Pen/Strep  OD: DMEM, 10% FBS, 50 μM ascorbate-2-phosphate, 10 mM βGP, 100 nM Dex | Polymer-based precipitation | 50, 100 µg/mL | RUNX2, ALP, COL1A1, OCN | TEM, NTA, WB, Cell scratch assay, qRT-PCR, ARS, PKH26 staining, CCK8 assay | Levels of 25 miRNAs in PDLSC-Exos in the 3D strain microenvironment (SM-Exo) were dramatically different from those obtained from the 3D culture microenvironment (Exo). |
| Lan et al. 2024 (68); China | Periodontitis | Premolars & 3^rd^ Molars; P3 | Cell culture: DMEM, 10% FBS, 1% Pen/Strep  OD: DMEM, 10% FBS, 50 μM ascorbate-2-phosphate, 10 mM βGP, 100 nM Dex | Polymer-based precipitation  Differential ultracentrifugation | 50, 100 µg/mL | CASPASE-3, BCL-2, BAX, APAF-1 | FCM, TEM, WB, CCK-8 assay, Cell scratch assay, ALP staining, ARS, qRT-PCR | PDLSC-Exo can promote hFOB1.19 cell proliferation, migration and OD, inhibiting H2O2-induced apoptosis, and activating the PI3K/AKT and MEK/ERK signaling pathways. |
| Li et al. 2024 (69); China | Orthodontically-induced inflammatory root resorption | Premolars; P3-P5 | Cell culture: DMEM, 20% FBS, 1% Pen/Strep  OD: DMEM, 10% FBS, 10 nmol/L Dex, 0.2 mmol/L ascorbate-2-phosphate, 10 mM βGP | Ultrafiltration & Differential Ultracentrifugation | 10, 50 µg/mL | RUNX2, OSX, OCN, CAP, COL1A1 | TEM, NTA, WB, qRT-PCR, CCK-8 assay, Cell scratch assay, ARS | PDLSC-Exos can promote the activity of cementoblasts via the PI3K/AKT signaling pathway |
| Wang et al. 2023 (70); China | Periodontitis | 3^rd^ Molars; P2-P4 | Cell culture: α-MEM, 10% FBS, 100 U/mL Pen, 100 mg/mL Strep  OD: NR | Differential ultracentrifugation | 5 µg/mL | MRC1, IL-10, IL-8, MINCLE, IL-12, CCR7, IL-1β | DLR assay, qRT-PCR, WB, FCM, TEM, CFU assay, CCK-8 assay | PDLSC-Exos in an inflammatory environment shuttled miR-143-3p, which targeted PI3Kγ in macrophages and promoted pro-inflammatory M1 macrophage polarization by suppressing PI3K/AKT signaling and activating NF-κB signaling. |
| Albougha et al. 2024 (71); Japan | Bone defects | Immortalized hPDL fibroblast cell line; NR | Cell culture: α-MEM, 10% FBS, 50 µg/mL Strep, 50 U/mL Pen  OD: NR | Polymer-based precipitation  Differential ultracentrifugation | 60 µg/mL | ALP, BMP2, OCN, OPN, RUNX2 | TEM, NTA, WB, Cell scratch assay, ARS, qRT-PCR | PDLSC-Exo promoted the migration, mineralization, and expression of ALP, BMP2, OCN, and OPN in human osteoblast-like cells, suggesting that PDLSC-Exos may be an attractive treatment tool for bone healing in defective periodontal tissue. |
