# Supplementary material for: Mechanistic insights into periodontal ligament stem cell-derived exosomes in tissue regeneration
Source: Clin Oral Investig. 2025 Jun 25;29(7):357. doi: 10.1007/s00784-025-06422-1 (PMC12198077; doi:10.1007/s00784-025-06422-1)
Supplement: Supplementary file 3 — Supplementary file3 (DOCX 27 KB) [file 784_2025_6422_MOESM3_ESM.docx]

**Table S3**. Primary characteristics of the included *in-vivo* studies.

| **Study & Country** | **Field (Disease)** | **Species & Weight** | **Age and sex** | **Sample size** | **Research Groups** | **Exosome Concentration** | **Assays** | **Main Findings** |
| --- | --- | --- | --- | --- | --- | --- | --- | --- |
| Lei et al. 2022 (32); China | Periodontitis | SD rats;  103-120 g | NR; Male | n=36 | (1) Defects left untreated (Control); (2) Defects treated with matrigel with Exos; and (3) Defects treated with Matrigel without Exos | 150000 μg/mL | μ-CT, H&E staining, Masson staining, IFS | Compared to the vehicle-treated control group, h-PDLSC-exosome-treated rats resulted in more bone formation in the defect of alveolar bone |
| Shi et al. 2023 (33); China | Periodontitis | C57BL/6J mice; NR | 6-week; Male | n=30 | (1) PBS for 5 mL (Control); (2) LPS (2 mg/mL LPS for 5 mL); (3) LPS+ASP^Low^ (60 ng/mL aspirin for 5 mL); (4) LPS+ASP^High^ (3 mg/mL aspirin for 200 mL), and (5) LPS+Exos (2 mg/mL Exos for 5 mL) and LPS+Exos-ASP (2 mg/mL EVs-ASP for 5 mL) | 2000 μg/mL | μ-CT, H&E staining, IFS | EVs-ASP inhibited the inflammatory environment of LPS-induced macrophage, and promoted anti-inflammatory macrophages formation, and reduced bone loss in periodontitis models. |
| Wang et al. 2023 (34); China | Periodontitis | SD rats; 150-180 g | NR; Male | n=18 | (1) Matrigel with Control Exos; (2) Exos overexpressed miR-200b; and (3) Exos overexpressed miR-200c | 1000 μg/mL | μ-CT, H&E staining, Masson staining, Immuno-histologic staining | Exos functionalized by mechano-responding miRNAs are effective components for bone regeneration under inflammatory conditions, providing a novel therapeutic opportunity for inflammatory bone loss in periodontitis. |
| Isik et al. 2023 (35); Turkey | Bone defects | Wistar rats; 300-500 g | 24-month; Female | n=24 | (1) Defect only (negative control); (2) Autograft (positive control); (3) GelMA (scaffold group without Exos); and (4) GelMA/hPDLFs-Exo (exosome inoculated scaffold group) | 100 μg/mL | μ-CT, H&E staining, qRT-PCR | Rats treated with GelMA/hPDLFs-Exos posed higher new bone mineralization compared to the negative controls |
| Liu et al. 2022 (36); Iraq | Orthodontic tooth movement | SD rats; 300±25 g | 6-8-week; Male | n=32 | (1) Saline (Control); (2) simvastatin in saline; (3) PDLSC-Exos; and (4) exosomal simvastatin | 4000 µg/mL | WB, H&E staining | Encapsulating simvastatin into PDLSC-Exos can improve simvastatin solubility and enhance the inhibition effect of relapse in the rat model of OTM; Local injection of PDLSC-Exos alone can also block the relapse after OTM |
| Tang et al. 2023 (37); China | Inflammatory bowel disease | BALB/c mice; NR | NR; NR | n=30 | (1) PBS (Control); (2) DSS; (3) DSS+ 37◦C-Exos; (4) DSS+43◦C-Exos; and (5) DSS+4◦C-Exos | 1000 µg/mL | H&E staining, WB, ELISA | EVs derived from thermally activated MSCs exhibited high expression of PD-L1, which modulated Th17/Treg cell differentiation through the PD-1/PD-L1 signaling pathway and played a significant anti-inflammatory role in DSS-induced colitis. |
| Huang et al. 2022 (39); China | Orthodontic tooth movement | C57BL/6J mice; 20-25g | 8-week; Male | NR | (1) PBS (Control); and (2) PDLSC-Exos in PBS | 1000 µg/mL | μ-CT, TRAP staining, qRT-PCR, IFS | The infusion of PDLSC-Exos into mice promotes mechanical force-induced tooth movement and increases osteoclasts in PDL. |
| Diomede et al. 2018 (30); Italy | Bone defects | Wistar rats; 300-350 g | NR; Male | n=24 | (1) Evo: scraping of cortical calvarium bone tissue and implant of Evo; (2) Evo+PDLSCs: scraping of cortical calvarium bone tissue and implant of Evo enriched with PDLSCs; (3) Evo+Exos: scraping of cortical calvarium bone tissue and implant of Evo enriched with Exos; (4) Evo+Exos+PDLSCs: scraping of cortical calvarium bone tissue and implant of Evo enriched with Exos+PDLSCs. | 500 µg/mL | IFS, PKH26 staining | Evo enriched with hPDLSCs and PEI-EVs can promote a bone regeneration process for the treatment of calvarium and ossification defects caused by accidental or surgery trauma. |
| Niu et al. 2024 (43); China | Periodontitis | C57BL/6J mice; NR | 6-8-week; Female | n=40 | (1) Ligated and PBS treatment (Induced); (2) Induced+Nor-Exos; (3) Induced+NC-Exos); and (4) Induced+FoxO1-Exos | 2000 µg/mL | μ-CT, TRAP staining, H&E staining, IFS, Bioluminescence imaging | FoxO1-overexpressed PDLSC-Exos effectively promoted bone formation and inhibited inflammation. |
| Pizzicannella et al. 2019 (44); Italy | Bone defects | Wistar rats; 300-350 g | NR; Male | n=20 | (1) 3D-COL: scraping of the cortical calvaria bone tissue and implant of 3D-COL; (2) 3D-COL/PDLSCs: scraping of the cortical calvaria bone tissue and implant of 3D-COL enriched with PDLSCs; (3) 3D-COL/PDLSCs/CM: scraping of the cortical calvaria bone tissue and implant of 3D-COL enriched with PDLSCs and CM; (4) 3D-COL/PDLSCs/Exos: scraping of the cortical calvaria bone tissue and implant of 3D-COL enriched with PDLSCs and Exos; and (5) 3D-COL/PDLSCs/PEI-Exos: scraping of the cortical calvaria bone tissue and implant of 3D-COL enriched with hPDLSCs and PEI-EVs | 50 µg/mL | IFS, μ-CT, H&E staining | 3D-COL enriched with hPDLSCs and PEI-EVs may promote bone regeneration of calvaria defects, associated also with an increased vascularization. |
| Zhang et al. 2020 (47); China | Periodontitis | SD rats; 200.4±25.3 g | NR; Female | n=20 | (1) N^Exos^: PDLSC-Exos from healthy subject (Control); (2) P^Exos^: PDLSC-Exos from periodontitis patients; and (3) (N+TNF-α 14d)^Exos^: PDLSC-Exos from healthy subjects treated with TNF-α for 14 days | 50000 µg/mL | IFS, μ-CT | The vascularization of PDL was upregulated in periodontitis, which might credit to the fact that PDLSCs regulated angiogenesis of HUVECs via exosome-mediated transfer of VEGFA targeted by miR-17-5p. |
| Zhao et al. 2022 (19); China | Alveolar bone regeneration | SD rats; NR | 8-week; Male | n=20 | (1) PBS/Matrigel (Control); and (2) 20 µL P-EV/Matrigel was plugged into one defect | 6000 µg/mL | μ-CT, H&E staining, Masson staining, IFS | P-EV/Matrigel accelerated bone tissue repair by increasing cell infiltration when compared with the control. |
| Lu et al. 2023 (48); China | Periodontitis | C57BL/6 mice; NR | 6-8-week; Male | n=24 | (1) Blank (control); (2) CP; (3) CP+NG-PDLSC-Exos; and (4) CP+HG-PDLSC-Exos | 15 µg/mL | MB staining, μ-CT, H&E staining, TRAP staining, qRT-PCR, DLR assay, WB | In experimental periodontitis, PDLSC-Exo reduced alveolar bone destruction and decreased the number of osteoclasts on the alveolar bone surface. |
| Kang et al. 2023 (50); China | Periodontitis | SD rats; 300±15 g | 8-week; Male | n=18 | (1) Normal saline (Control); (2) LPS: 10 μl *E. coli* LPS; and (3) LPS+Exos‐miR‐205‐5p: 10 μg Exos isolated from miR‐205‐5p mimics‐transfected PDLSCs | 100 µg/mL | H&E staining, FCM, qRT-PCR, ELISA, DLR assay, WB | Exo‐miR‐205‐5p weakens the inflammation of CP in a rat model via inhibiting the production of inflammatory factors and the imbalance of Th17/Treg cells. |
| Wu et al. 2023 (53); China | Periodontitis | SD rats; NR | 6-8-week; Male | n=20 | (1) Control: macrophages cultured in Exos-free DMEM without transfection; (2) Exos: Exos from 6-h stretched PDLSCs and administered to macrophages at 100 μg/mL; (3) Inhibitor: 100 μg/mL Exos were transfected into macrophages for 24 h; and (4) Inhibitor-NC: 100 nM has-miR-9-5p were transfected into macrophages for 24 h. | 100 µg/mL | DLR assay, H&E staining, IFS, IHC | PDLCs could transmit the mechanobiological signals to immune cells by releasing Exos and simultaneously enhance periodontal inflammation through the miR-9-5p/SIRT1/NF-kB pathway. |
| Zhao et al. 2022 (56); China | Bone defects | SD rats; 250-300 g | NR; Male | n=9 | (1) PBS (control); (2) Gel-Alg hydrogel; and (3) Hydrogel+Exos | 2000 µg/mL | μ-CT, H&E staining, Masson staining | Compared with the control group and the hydrogel group, the rats in the hydrogel with Exos group showed more new bone formation in alveolar bone defects. |
| Lu et al. 2023 (59); China | Periodontitis | SD rats; 200-250 g | 8-week; Male | n=20 | (1) EV group: 20 μg P-EVs (in 10 μL PBS)+10 μL Matrigel; and (2) Control: Mixed PBS+Matrigel | 2000 µg/mL | H&E staining, Masson staining, IHC | PDLSC-Exos induced bone regeneration in a calvarial defect model. |
| Soundra Rajan et al. 2017 (60); Italy | Multiple sclerosis | C57BL/6 mice; 20-25 g | 12-week; Male | n=30 | (1) Naïve: normal mice with no (MOG) 35–55 or other immunization; (2) EAE: mice subjected to EAE with no other treatment; (3) EAE+hPDLSCs-CM; (4) EAE+hPDLSCs-EMVs; (5) naïve+hPDLSCs-CM; and (6) naïve+hPDLSCs-EMVs | 24 µg/mouse | H&E staining, IHC, WB | The study showed enhanced expression of NALP3, Cleaved Caspase 1, IL-1β, and IL-18 in EAE mouse spinal cord; PDLSCs-conditioned medium and Exos significantly blocked NALP3 inflammasome activation and provided protection from EAE. |
| Rajan et al. 2016 (61); Italy | Multiple sclerosis | C57BL/6 mice; 20-25 g | 12-week; Male | n=30 | (1) Naive group; (2) EAE group; (3) EAE+hPDLSCs-CM from RR-MS patients; (4) EAE+hPDLSCs-CM from healthy; (5) EAE+hPDLSCs-EMVs from RR-MS; (6) EAE+hPDLSCs-EMVs from healthy; (7) NAIVE+hPDLSCs-CM from RR-MS; (8) NAIVE+hPDLSCs-CM from healthy; (9) NAIVE+hPDLSCs-EMVs from RR-MS; and (10) NAIVE+hPDLSCs-EMVs from healthy | 24 µg/mouse | H&E staining, IHC, ELISA, WB | The study unravel the immunosuppressive effects of hPDLSCs-CM and hPDLSC-Exos in EAE mice, and suggest simple alternative autologous source for patient-customized cell-free targeting treatment in MS patients. |
| Xu et al. 2022 (65); China | Orthodontic tooth movement | C57BL/6 mice; NR | 8-week; Male | n=24 | (1) OTM; (2) OTM+normal saline; and (3) OTM+Exos | 1 µg/mL | μ-CT, IHC, H&E staining | PDLSC-Exos effectively enhanced OTM and promoted osteogenesis on the tension side, including increasing trabecular bone parameters and promoting the expression of osteogenic-related biomarkers |
| Zhong et al. 2022 (66); China | Diabetes Mellitus | SD rats; NR | 8-week; Male | n=6 | (1) Control: Untreated HA coated with PLL and CMC; (2) Defect: Periodontal defect in experimental model; (3) HA; (4) HALL: HA coated with PLL and CMC; and (5) Met@HALL: Met-loaded HA coated with PLL and CMC | NR | μ-CT, H&E staining, Masson staining, IFS | Compared to controls, Met@HALL with enhanced cytocompatibility and pro-osteogenic activity could boost the remodeling of diabetic periodontal tissue in rats. |
| Yu et al. 2021 (67); China | Alveolar bone regeneration | SD rats; 150-180 g | NR; Male | n=24 | (1) Matrigel; (2) Exos/Matrigel; (3) SM-Exos/Matrigel; and (4) PBS (Control) | 1000 µg/mL | μ-CT, H&E staining, Masson staining, IHC | PDLSC-Exos showed stronger bioactivity to repair alveolar bone defects in SD rats |
| Wang et al. 2023 (70); China | Periodontitis | C57BL/6 mice; NR | 8-week; Male | n=24 | (1) Sham+PBS@NC-Exos; (2) Ligature+PBS@NC-Exos; (3) Ligature+PBS@Mimic-Exos; and (4) Ligature+740Y-P@Mimic-Exos | 3000 µg/mL | H&E staining, IFS | Exos-shuttled miR-143-3p from PDLSCs drove M1 macrophage polarization and aggravated periodontal inflammation in a mouse periodontitis model. |
| Albougha et al. 2024 (71); Japan | Bone defects | SD rats; 300-350 g | 12-week; Male | n=6 | (1) PDLSC-Exos+VitroGel; and (2) PBS+VitroGel (Control) | 60 µg/mL | μ-CT, H&E staining, Masson staining | More newly formed bone was observed in PDLSC-Exos-treated group than in the non-treated group at the defect sites in rats. |
